# Assessment of Accelerated Aging Effect of Bio-Oil Fractions Utilizing Ultrahigh-Resolution Mass Spectrometry and k-Means Clustering of van Krevelen Compositional Space

**DOI:** 10.1021/acs.energyfuels.4c02605

**Published:** 2024-08-20

**Authors:** Diana Catalina Palacio Lozano, Daniel W. Lester, J.S. Town, Amy M. McKenna, Martin Wills

**Affiliations:** † Department of Chemistry, University of Warwick, Coventry CV4 7AL, U.K.; ‡ Polymer Characterisation Research Technology Platform, University of Warwick, Coventry CV4 7AL, U.K.; § National High Magnetic Field Laboratory, Florida State University, 1800 East Paul Dirac Drive, Tallahassee, Florida 32310-4005, United States; ∥ Department of Soil and Crop Sciences, Colorado State University, Fort Collins, Colorado 80523, United States

## Abstract

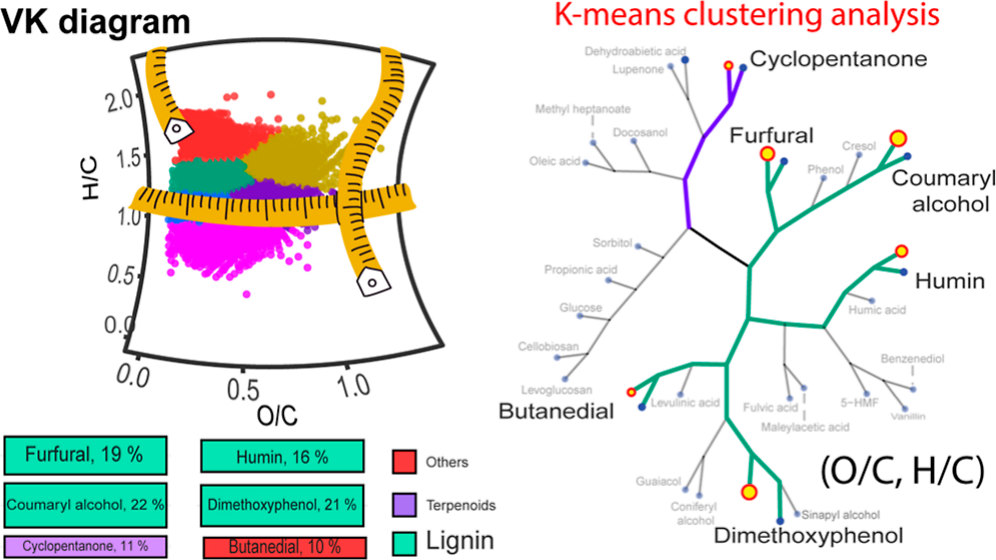

Bio-oils contain a substantial number of highly oxygenated hydrocarbons, which often exhibit low thermal stability during storage, handling, and refining. The primary objectives of this study are to characterize the hydroxyl group in bio-oil fractions and to investigate the relationship between the type of hydroxyl group and accelerated aging behavior. A bio-oil was fractionated into five solubility-based fractions, classified in two main groups: water-soluble and water-insoluble fractions. These fractions were then subjected to chemoselective reactions to tag molecules containing hydroxyl groups and analyzed by negative-ion electrospray ionization 21 T Fourier transform ion cyclotron resonance mass spectrometry (FT-ICR MS). The fractions were also subjected to accelerated aging experiments and characterized by FT-ICR MS and bulk viscosity measurements. Extracting insightful information from ultrahigh-resolution data to aid in predicting upgrading methodologies and instability behaviors of bio-oils is challenging due to the complexity of the data. To address this, an unsupervised learning technique, k-means clustering analysis, was used to semiquantify molecular compositions with a close Euclidean distance within the (*O*/*C*, *H*/*C*) chemical space. The combination of k-means analysis with findings from chemoselective reactions allowed the distinctive hydroxyl functionalities across the samples to be inferred. Our results indicate that the hexane-soluble fraction contained numerous molecules containing primary and secondary alcohols, while the water-soluble fraction displayed diverse groups of oxygenated compounds, clustered near to carbohydrate-like and pyrolytic humin-like materials. Despite its high oxygen content, the water-soluble fraction showed minimal changes in viscosity during aging. In contrast, a significant increase in viscosity was observed in the water-insoluble materials, specifically, the low- and high-molecular-weight lignin fractions (LMWL and HMWL, respectively). Among these two fractions, the HMWL exhibited the highest increase in viscosity after only 4 h of accelerated aging. Our results indicate that this aging behavior is attributed to an increased number of molecular compositions containing phenolic groups. Thus, the chemical compositions within the HMWL are the major contributors to the viscosity changes in the bio-oil under accelerated aging conditions. This highlights the crucial role of oxygen functionality in bio-oil aging, suggesting that a high oxygen content alone does not necessarily correlate with an increase of viscosity. Unlike other bio-oil categorization methods based on constrained molecule locations within the van Krevelen compositional space, k-means clustering can identify patterns within ultrahigh-resolution data inherent to the unique chemical fingerprint of each sample.

## 1 Introduction

**Figure 1:**
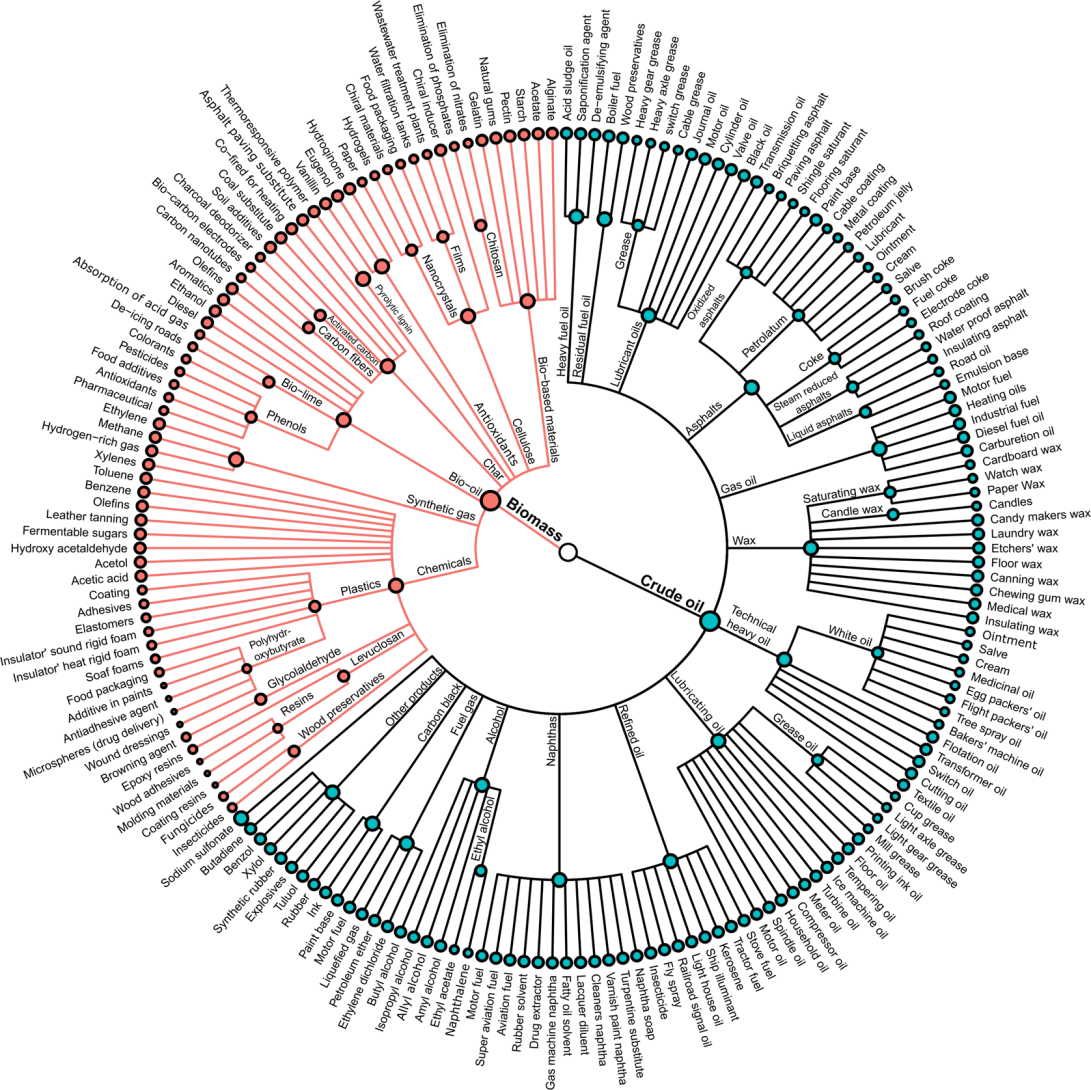
Dendrogram illustrating crude oil’s byproducts, adapted from Socony-Vacuum Oil Company’s product tree.^17^ Potential biobased materials are illustrated in red.^2−4^

Diversifying the chemical industry’s resources beyond fossil materials by utilizing renewable sources aligns with the principles of Green Chemistry.^1^ As presented in Figure 1, biomass can potentially be refined into a wide spectrum of products.^2−4^ Unfortunately, biomass refining is not as advanced or efficient as the refinement of crude oils.^5^ Thermochemical processes such as pyrolysis, gasification, and liquefaction are necessary to transform biomass materials into a diverse range of byproducts.^6,7^ The final products derived from pyrolysis include a liquid bio-oil containing thousands of organic chemical compositions including aromatic products known as lignin and carbohydrate polymers (cellulose and hemicellulose).^4^

Bio-oils, unlike crude oils, contain a significant amount of highly oxygenated hydrocarbons,^8−10^ often displaying low thermal stability during storage, handling, and refining. The instability of bio-oil is commonly referred to as “aging”, which is defined as the process of irreversible properties changing over time. The aging of bio-oil involves combined polymerization of furans, esterification of alcohols with organic acids, hydration of aldehydes or ketones with water, acetylation, and other reactions, the full extent of which remains incompletely understood.^11,12^ Aging processes can significantly alter the chemical and physical properties of bio-oils.^13^ For instance, Boucher et al.^14^ found that the viscosity of bio-oil increases dramatically when it is stored at room temperature for 65 days. Phase separation also occurs in aged bio-oil as a consequence of increased water content during storage.^13^ Changes in the chemical composition of aged bio-oil include a reported decrease in aldehydes, ketones, carbonyls,^15^ and carbohydrate compounds and a decrease in high-volatile chemicals containing phenolic hydroxyl and methoxy groups in pyrolytic lignin^13,16^

Aging tests such as accelerated aging, in which the samples are exposed to high temperatures, are common methods used to measure bio-oil stability, especially to track changes in bio-oil viscosity. Standard analytical techniques such as gas chromatography, total acid number determination, elemental analysis, viscosity measurement, water content assessment, and acidity are typically employed to characterize the physical and chemical properties of bio-oil and to study the effects of aging. However, due to the inherent complexity of bio-oils, these methods encounter limitations, particularly in achieving precise chemical composition separation and accessing nonvolatile chemicals with a high degree of detail.

Fourier transform ion cyclotron resonance mass spectrometry (FT-ICR MS) has demonstrated the unique ability to resolve species from one another within complex background sample matrices and can distinguish molecular species that differ in mass by the mass of an electron.^18−23^ Thus, FT-ICR MS is particularly suitable for the analysis of renewable complex mixtures, such as bio-oils. FT-ICR MS provides sub-ppm mass accuracy for species that contain carbon and hydrogen atoms and other heteroatoms. The compositions are expressed as molecular formulas in the form C_c_H_h_N_n_O_o_S_s_ (where c, h, n, o, and s represent the number of carbon, hydrogen, nitrogen, oxygen, and sulfur atoms, respectively). The molecular compounds are sorted according to the heteroatomic class, double-bond equivalents (DBE), calculated from the elemental composition, atomic hydrogen-to-carbon ratio (*H*/*C*), and oxygen-to-carbon ratio (*O*/*C*) for both visualization and analysis. However, all mass spectrometry techniques group isomeric species into one mass-to-charge ratio, and complex organic mixtures contain multiple isomeric species that cannot be differentiated by direct infusion analysis alone.^24,25^ For instance, a compound assigned with a molecular formula of C_9_H_12_O_2_ might correspond to 4-ethyl-2-methoxyphenol or conversely to any other chemical sharing the same mass-to-charge ratio. With over 3300 distinct chemicals reported in databases such as ChemSpider,^26^ all having a molecular formula of C_9_H_12_O_2_, it is evident that novel analytical methods are necessary to differentiate or discern isomeric compositions within complex mixtures to highlight structural diversity.

Elucidating structural characteristics and their possible role in specific bio-oil properties and behaviors upon storage and upgrading can be the key in finding efficient bio-oil upgrading routes and understanding aging mechanisms. Thus, more research is needed to understand the effect of the presence of a functional group in macro properties such as viscosity and storage stability.

Pyrolysis bio-oils contain thousands of oxygenated compounds with a wide range of chemical groups and different polarities. Separation methods such as solvent extraction use differences in polarity to effectively separate bio-oil’s constituents into distinct fractions.^27^ Additionally, many researchers have employed offline fractionation for bio-oils prior to FT-ICR MS analysis.^28−31^ These separation techniques are crucial not only for enhancing the analytical understanding of the molecular diversity of bio-oils^32,33^ but also serve as a pivotal pretreatment stage for biorefineries, aimed at enhancing the overall quality of biobased products. Chemoselective reactions prior to mass spectrometry detection can also be used to enhance the chemical characterization of a complex sample. Palacio Lozano et al.^34^ recently proposed a chemoselective derivatization method in combination with FT-ICR MS with the aim of tagging compositions containing hydroxyl groups in complex mixtures. The increased number of the O_o_S_s_ heteroatomic class after the derivatization in DMSO-Ac_2_O was clear evidence of the isomeric diversity of bio-oils. Thus, chemoselective reactions can be used to typify functional groups in a complex mixture, providing unique structural characteristics.

The combination of FT-ICR MS with chemoselective reactions adds another dimension to MS data by providing structural characteristics. This makes meaningful data interpretation challenging when using traditional visualization tools, such as class distribution, DBE, and van Krevelen (VK) diagrams. Classification of molecular compositions into chemical categories has emerged as a vital technique for FT-ICR MS’ data interpretation. For instance, Hockaday et al.^35^ proposed a classification system encompassing pyrolytic lignin-like, carbohydrate-like, protein-like, lipid-like, unsaturated hydrocarbon-like, and condensed aromatic-like structures. This characterization is based on ranges of *H*/*C* and *O*/*C* (VK compositional space), which are characteristic of well-known molecules in natural product literature. Similar classification frameworks have been proposed by Olliver et al.,^36^ Rivas-Ubach et al.,^37^ and Brockman et al.,^38^ among others. These classifications aim to uncover properties or trends of data that often comprise thousands of molecular compositions, thereby providing valuable insights into molecular reactivity, storage stability, environmental impact, and more. However, classification schemes applied to VK diagram areas can vary widely across literature sources, contributing to potential inconsistencies and inaccuracies in compound classification.

Statistical tools could be used as an alternative method to help find correlations among large sets of complex data at the molecular level.^39^ When data objects’ features (e.g., chemical identification) are unknown, appropriate classification of the data can be handled by cluster analysis.^40−42^ One of the most common types of cluster analysis is k-means. The goal of clustering analysis is to group data objects whose attributes are similar together in a cluster, so that the similarity of the data within the cluster is higher when compared with other cluster’s data objects. In contrast with the current molecular composition classification of bio-oils, k-means analysis could offer an unsupervised method to identify latent patterns and groupings in a large data set.

The aim of this study is to typify the hydroxyl group in bio-oil fractions by using DMSO-AC_2_O reactions in combination with 21 T FT-ICR MS and to gain insights into the effect of this functional group during accelerated aging experiments. We also propose using k-means clustering analysis of the VK compositional space to classify complex data sets generated by FT-ICR MS.

Unlike alternative methods based on the VK compositional space, k-means clustering is inherent to the specific compositional characteristics of the samples, providing a flexible approach for semiquantifying the total number of molecules within each cluster. This approach allowed us to infer the presence of a greater number of oxygenated compounds containing primary and secondary alcohols in the hexane-soluble fraction, while the water-soluble fraction exhibited diverse oxygenated compounds grouped close to carbohydrate-like and pyrolytic humin-like materials. Future studies, such as tandem mass spectrometry, are recommended to investigate the structural characteristics of the molecular compositions in greater detail within these VK compositional spaces. Despite its high oxygen content, the water-soluble fraction showed minimal viscosity changes during accelerated aging. Furthermore, two water-insoluble lignin fractions, low-molecular-weight lignin (the fraction soluble in dichloromethane, known as LMWL) and high-molecular-weight lignin (the fraction insoluble in dichloromethane, known in the literature as HMWL), displayed contrasting behaviors, with HMWL transforming into a highly viscous material within 4 h of aging, attributed to its increased phenolic group content. Thus, our results suggest that the phenolic compounds present in the HMWL have a detrimental effect on bio-oil aging. These findings highlight the significance of oxygen functionality diversity and the impact of functional groups on bio-oil aging and storage.

## 2 Experimental Section

### 2.1 Bio-Oil Fractionation

**Scheme 1:**
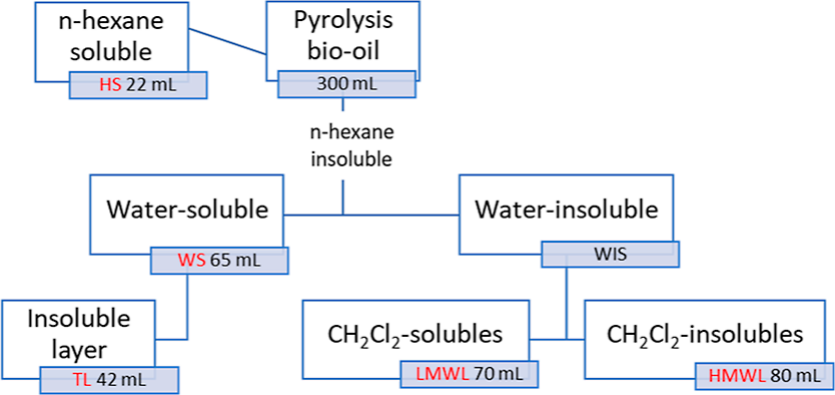
Solvent Fractionation Scheme into HS (Hexane-Soluble), Water-Soluble (WS), LMWL, and HMWL

A bio-oil was purchased from BTG BioLiquids (Enschede, The Netherlands). In brief, the bio-oil is produced from the fast pyrolysis of lignocellulosic material (wood-derived bio-oil) with a short vapor resident time at 450–600 °C, near atmospheric pressure in the absence of oxygen. The bio-oil was subsequently separated into distinct subfractions following the approach outlined by Oasmaa et al.^43,44^ In brief, 300 mL of bio-oil was mixed with 100 mL of *n*-hexane and stirred continuously for 2 h. This process was repeated four times, with an additional 100 mL of *n*-hexane added each time (400 mL of *n*-hexane was added in total). This fraction was then vacuum-rotary-evaporated at 40 °C to a final volume of approximately 22 mL. The *n*-hexane-insoluble fraction was further separated in two fractions: water-soluble (WS) and water-insoluble (WIS) fractions by adding a total of 750 mL of ice-cooled water in 100 mL batches, with each batch stirred for 2 h. A heavy tar-like material settled at the bottom of the flask, while the water-soluble material formed the top liquid layer. The top layer was carefully extracted and filtered with a Whatman no. 4 filter to remove any remaining solids and was left to dry, yielding a final volume of about 65 mL. Additionally, a top layer was observed above the water-soluble fraction, named hereafter the TL-fraction; this fraction was separated using a separation funnel. Subsequently, the WIS fraction was further separated into dichloromethane-soluble (referred to in the literature as LMWL) and dichloromethane-nonsoluble fractions (referred to in the literature as HMWL). The LMWL fraction was extracted with a glass pipet, and the solvent was vacuum-rotary-evaporated at 40 °C to a final volume of 70 mL. The HMWL fraction was extracted with methanol and similarly evaporated on a rotary evaporator to a final volume of 80 mL. Scheme 1 illustrates this fractionation process.

### 2.2 Acetylation Reactions

Following the separation process, each fraction was prepared according to the method described by Palacio Lozano et al.^34^ In brief, two sets of each fraction were prepared as follows: 80 mg of the bio-oil fractions was added to two vials, with one set of each fraction being diluted in 1 mL of HPLC-grade dimethyl sulfoxide (DMSO, Sigma-Aldrich, Gillingham, United Kingdom), while the other set was diluted in a mixture of 300 mL of acetic anhydride (Ac_2_O, 99%, Fischer Scientific, Loughborough, UK) and 700 mL of DMSO. The vials were allowed to react at room temperature for approximately 7 days, after which they were stored at −23 °C until FT-ICR MS analysis. The derivatized samples are hereafter labeled with DMSO-Ac_2_O for identification purposes.

### 2.3 Accelerated Aging Test

The aging tests were performed for the fractions with the highest yields, that is, WS, LMWL, and HMWL. The test is described as follows: 60 mL of each fraction was added to a 250 mL round-bottom flask equipped with a reflux condenser, a thermometer, and a mechanical stirrer and was then exposed to a temperature of 80 °C.^11,45^ The reflux condenser was used to prevent the contents of the bottom flask from undergoing complete evaporation. Approximately 5 mL of each fraction was extracted at regular intervals, as detailed in Table 1. Extraction concluded upon observation of a significant change in the viscosity properties of the fractions. Notably, this alteration was observed in the HMWL fraction after 4 h of accelerated aging, resulting in the collection of only five aging time points for this sample. Rheology and FT-ICR MS analyses were then performed as described below.

**Table 1:** List of Aging Tests Performed for the WS and the LMWLL and HMWL Fractions, Respectively

aging test (h)		
WS	LMWL	HMWL
0	0	0
0.5	0.5	0.5
1	1	1
2	2	2
4	4	4
6	6	
11	11	
24	17	
34	24	
50	41	

### 2.4 Negative-Ion 21 T ESI FT-ICR MS Analysis

The bio-oil fractions, acetylated fractions, and aging test samples were analyzed with a custom-built hybrid linear ion trap FT-ICR mass spectrometer equipped with a 21 T superconducting solenoid magnet.^46,47^ The samples were diluted in MeOH (100%) to a final concentration of 100 μg/mL and directly infused via a microelectrospray source (50 μm i.d. fused silica emitter) at 500 nL/min by a syringe pump.^48^ Typical conditions for negative-ion formation were: emitter voltage, −2.8–3.2 kV; S-lens RF level, 40%; and heated metal capillary temperature, 350 °C. The ions were initially accumulated in an external multipole ion guide (1–5 ms) and released *m*/*z*-dependently by a decrease of an auxiliary radio-frequency potential between the multipole rods and the end-cap electrode.^49^ Ions were excited to *m*/*z*-dependent radius to maximize the dynamic range and number of observed mass spectral peaks (32–64%), and excitation and detection were performed on the same pair of electrodes.^49,50^ FT-ICR MS detection window was optimized for the detection of ions with *m*/*z* in between 150 and 1800. The dynamically harmonized ICR cell in 21 T FT-ICR is operated with a 6 V trapping potential.^49,51^ Hundred individual time-domain transients with AGC ion target of 2 × 10^6^ charges per scan of 3.1 s were conditionally coadded and acquired with the Predator data station that handled excitation and detection only, initiated by a TTL trigger from the commercial Thermo data station, with 100 time-domain acquisitions averaged for all experiments.^52^ Mass spectra were phase-corrected^53^ and internally calibrated with 10–15 highly abundant homologous series that span the entire molecular weight distribution based on the “walking” calibration method.^54^ Experimentally measured masses were converted from the International Union of Pure and Applied Chemistry mass scale to the Kendrick mass scale for the rapid identification of homologous series for each heteroatom class.

Peaks with the signal magnitude greater than 4 times (4σ) the baseline root-mean-square (rms) noise at *m*/*z* 400 were exported to peak lists, and molecular formula assignments were performed with PetroOrg© software.^18,55^ Molecular formula assignments with error >0.5 parts per million were discarded, and only chemical classes with a combined relative abundance of ≥0.15% of the total were considered. For all mass spectra presented herein, 6800–38,000 peaks were assigned elemental compositions with the root-mean-square mass measurement accuracy of 57–98 ppb with the achieved resolving power of 3,300,000 at *m*/*z* 200 (time-domain detection of 3.1 s) and achieved resolving power of 1,800,000 at *m*/*z* 400. For each elemental composition C_c4–c500_H_h6–h200_N_n0–n4_O_o0–o40_S_s1–s5_, the heteroatom class, DBE (DBE = *C* – *h*/2 + *n*/2 + 1), hydrogen-to-carbon ratio (*H*/*C*), and oxygen-to-carbon ratios (*O*/*C*) were tabulated for subsequent data visualization using R scripts. Hereafter, (*O*/*C*, *H*/*C*) values will be used to define the chemical compositional space of a molecular composition. The mean oxygen content was calculated using the following equation.^56^
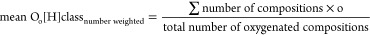
1

The number-average and weight-average molecular weights (Mn and Mw) calculated based on the FT-ICR-detected signal were calculated using eqs 2 and 3
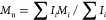
2
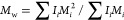
3where *I*_*i*_ and *M*_*i*_ represent the relative intensity and the *m*/*z* value of each molecular composition.

### 2.5 Rheology

The rheological properties of the aging test samples were observed on an Anton Paar MCR 382 (Anton Paar, Graz, Austria) rheometer equipped with parallel-plate geometry. A strain sweep was performed to identify the linear viscoelastic region of all samples with an angular frequency of 100 rads s^–1^. The complex viscosity recorded in this paper was taken from 0.5% strain as this was within the linear viscosity region of all samples. The viscosity was measured at 23 °C.

### 2.6 k-Means Clustering Analysis

k-Means clustering analysis, also called unsupervised learning, was used to perform cluster analysis by using FT-ICR MS data. The clustering analysis was performed using the compositional space determined by the *H*/*C* and *O*/*C* ratios of the monoisotopic molecular compositions. To simplify the analysis, nitrogen-containing assignments were excluded from clustering analysis. The data sets were imported into RStudio version 2023.12.1 + 402. Subsequent analyses were conducted using the “factoextra” package.^57−59^ This analysis encompassed the determination of the optimal number of clusters using the elbow method. The k-means Hartigan-Wong algorithm^60^ was employed for clustering. Additionally, cluster size (number of assignments per cluster) and cluster centers (*O*/*C*, *H*/*C*) were extracted.

Followed by this analysis, the FT-ICR MS cluster centers were hierarchically compared to the chemical space, (*O*/*C*, *H*/*C*) values, of chemicals that have been identified in the bio-oil literature.^43,61−63^ A detailed list of these standards is listed in Table S1 in the Supporting Information. Ward’s minimum variance method, ward.D2, in package “stats”, was used for this purpose.^64^ The hierarchy visualization was performed as dendrograms and k-means trees through the utilization of the “fviz_dend” function, as provided by the factoextra package. Statistical results are provided in the Supporting Information.

## 3 Results and Discussion

### 3.1 Molecular Characterization of Bio-Oil Fractions

FT-ICR MS instruments provide the most detailed molecular characterization of complex mixtures. A typical bio-oil’s mass spectrum contains thousands of highly oxygenated organic compounds. Graphical representations of such complex data sets include heteroatomic class distributions, double bond versus carbon number plots, and VK diagrams. Among these, VK diagrams are more commonly used when representing and interpreting the chemical space of highly oxygenated samples.^37,38,55,65,66^

The following section aims to outline the main chemical compositions and chemical spaces detected before and after acetylation reactions.

#### 3.1.1 Elemental Composition Profile of the Bio-Oil Fractions and Their DMSO-Ac_2_O Derivatives

Derivatization, as an additional step in sample preparation, has been used to improve the ionizability and detection of thiols,^67^ to characterize molecular compositions containing ketone/aldehyde functionalities,^68^ for semitargeted analysis of carbonyl compounds in bio-oils^69^ and for semitargeted analysis of the hydroxyl group.^34^ Thus, derivatization steps aim to tag functional groups to improve ionizability or to infer structural information. Structural information is typically limited by traditional MS acquisition methods that do not involve fragmentation or chromatography for isomeric separation.

**Figure 2:**
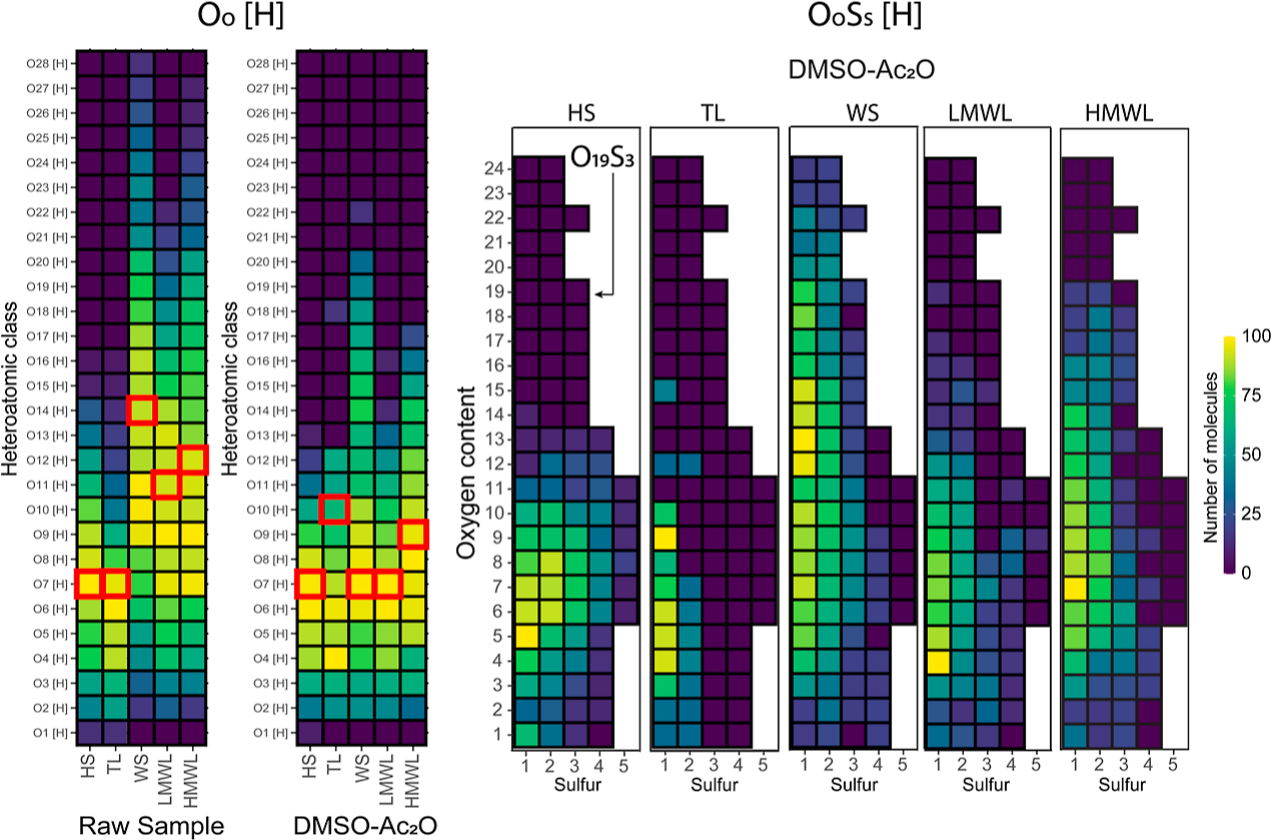
O_o_[H] and O_o_S_s_[H] heteroatomic class distribution of the bio-oil fractions before (raw sample) and after the reaction in DMSO-Ac_2_O mixtures. A red square marks the mean oxygen value. O_o_S_s_[H] compositions are represented as follows: sulfur atoms on the *x*-axis and oxygen atoms on the *y*-axis (see, e.g., the tile representing molecules corresponding to O_19_S_3_).

Semitargeted analysis of the hydroxyl group can be performed by preparing samples in a mixture of DMSO-Ac_2_O before direct infusion analysis. DMSO-Ac_2_O mixture, also known as the Albright-Goldman reagent, can be used for the oxidation of primary and secondary alcohols to aldehydes and ketones, respectively.^70^ Additionally, sterically hindered alcohols can lead to the formation of *O*-methylthiomethyl (*O*-*MTM*) byproducts,^71^ and phenols can lead to the attachment of MTM (*MTM* attachment) in an available ortho, para, or meta position in the benzene ring.^34,72^ Given the ultrahigh resolving power provided by FT-ICR MS, the MTM attachment can be detected and unambiguously assigned to O_o_S_s_[H] heteroatomic classes, allowing for the inference of a detailed structural arrangement of hydroxyl groups in complex mixtures.^34^ The results of DMSO-Ac_2_O reactions of the bio-oil fractions are presented in Table 2, while the heteroatomic class distribution is illustrated in the tile plot in Figure 2.

**Table 2:** Number of Assignments before and after Derivatization in DMSO-Ac_2_O Mixtures^a^

	group	HC	R-HC	TL	R-TL	WS	R-WS	LMWL	R-LMWL	HMWL	R-HMWL
number of assignments	O_o_[H]	15,272	9204	9607	3801	15,328	10,070	17,934	8411	19,712	7285
	O_o_S_s_[H]	NA	25,172	NA	2467	NA	18,169	NA	12,767	NA	13,828
	N_n_O_o_[H]	2417	1757	1591	543	2559	1276	1781	1983	4162	1928
	N_n_O_o_S_s_[H]	NA	1867	NA	0	NA	351	NA	434	NA	275
assignments [%]	O_o_[H]	86.3	24.2	85.8	55.8	85.7	33.7	91	35.6	82.6	32
	O_o_S_s_[H]	NA	66.2	NA	36.2	NA	60.8	NA	54.1	NA	60.8
	N_n_O_o_[H]	13.7	4.6	14.2	8	14.3	4.3	9	8.4	17.4	8.5
	N_n_O_o_S_s_[H]	NA	4.9	NA	0	NA	1.2	NA	1.8	NA	1.2
ratio	Oo[H]	0.6	NA	0.4	NA	0.7	NA	0.5	NA	0.4	NA
total assignments		17,689	38,000	11,197	6811	17,887	29,866	19,715	23,595	23,874	22,755

a Ratios are calculated as the number of compositions in DMSO-Ac_2_O/raw samples. NA: not applicable, not detected compositions.

Consistent with previous studies conducted on similar bio-oil fractions,^62^ the raw samples (before reaction) are primarily composed of highly oxygenated molecular compounds (>82%), followed by N_n_O_o_[H]. The WS fraction exhibited the highest mean oxygen content at O_14_[H], followed by the HMWL and LMWL fractions with a mean oxygen content of O_12_[H] and O_11_[H], respectively. Conversely, the HS and TL fractions are characterized by their lower oxygen-containing compounds with the lowest mean oxygen content recorded at O_7_[H]. In general, a decrease in the mean oxygen content was observed after the reactions in DMSO-Ac_2_O, particularly noticeable in the WS fraction, where the mean oxygen content was halved. This indicates that acetylation reactions were less favorable than DMSO-Ac_2_O reactions.

After the reactions, thousands of molecular compositions corresponding to the species of O_0_S_s_[H] were observed. Particularly noteworthy, the HS sample exhibited the highest number of compositions postreaction, including the highest number of MTM attachments in a single molecular composition, up to (CH_2_SCH_3_)5. Conversely, the TL fraction, characterized by lower reactivity in the DMSO-Ac_2_O mixture, presented just under 2500 O_o_S_s_[H] assignments, majority of which correspond to mono-MTM attachments (O_o_S_1_[H]). In contrast, over 18,000 O_o_S_s_[H] molecular compositions were assigned to DMSO-Ac_2_O WS, including MTM attachments as high as O_24_S_1–2_. This suggests a favorable trend toward mono- and dimethylthiomethylation in molecules rich in oxygen atoms within this sample. Similar results were observed for the HMWL fractions, albeit with a higher number of O_o_S_s_[H] molecules at a lower oxygen content.

**Figure 3:**
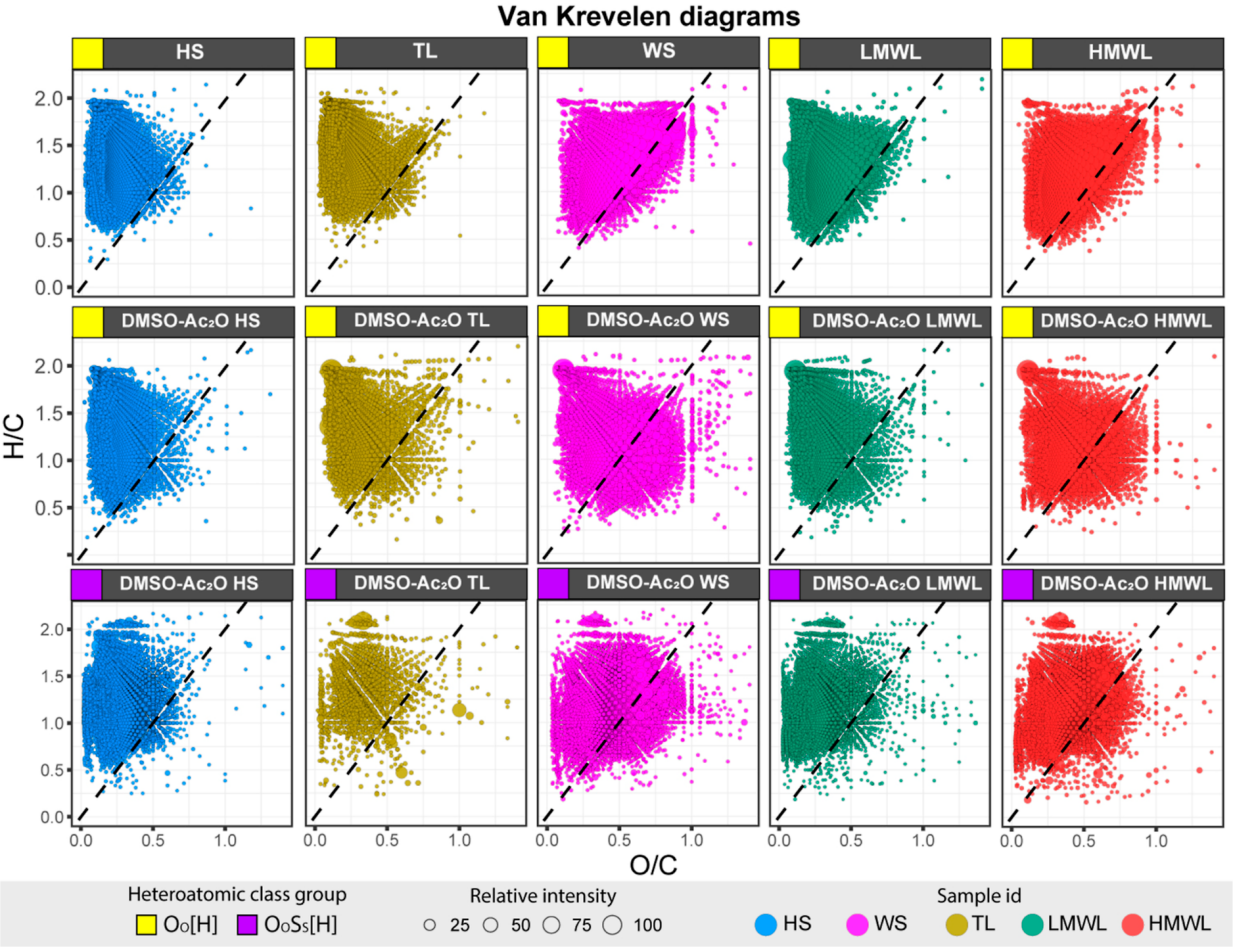
VK diagrams of the bio-oil fractions. The dehydration line (black dotted line) with slope −2 and intercept (0,0) was added as a reference line in each diagram. O_o_[H] heteroatomic classes are represented in the diagrams, with a yellow square in the top-left corner of each diagram, whereas a purple square is used to represent O_o_S_s_[H] heteroatomic classes.

VK diagrams of the bio-oil fractions are illustrated in Figure 3. The hydration line, characterized by a slope of 2, was included in the VK diagrams as a reference line to facilitate visual comparisons of compositional spaces across samples. VK diagrams can be used to represent the compositional space, revealing distinct characteristics among the various samples. For instance, the TL and HS samples are positioned in the region of the lowest unsaturation (higher *H*/*C* value) and low *O*/*C,* consistent with prior discussions on heteroatomic distribution. However, it is noteworthy that the TL fractions showed significantly lower reactivity compared to the HS fraction in DMSO-AC_2_O mixtures, suggesting a unique oxygen group profile. Given the TL fraction’s low reactivity, and the observed compositional space on the VK diagram, we hypothesize that this fraction contains the fewest number of hydroxyl groups. Chemical compositions in the TL fraction likely correspond to waxes, hydrophobic materials including long-chain alcohols, fatty acids, and fatty acid esters.

The HS fraction was particularly susceptible to MTM attachments. The O_o_S_s_[H] heteroatomic classes indicate MTM attachments either to an available ortho-para-meta position on a benzene ring or to an *O*-MTM attachment. *O*-MTM products are typically observed in alkaloids, carbohydrates, and steroids. Oasmaa et al.,^43,44^ and more recently Ohra-aho et al.,^73^ have demonstrated that the HS fraction is composed of lipophilic extractives such as fatty acids, steryl esters and sterols, terpenoids, and waxes. An *O*-MTM derivative is formed by the addition of CH_2_SCH_3_ on a hydroxyl position.^70^ Consequently, the *O*-MTM derivatives of sterols and terpenoids are suspected to be produced in this fraction. The primary and secondary OH groups in sterols, terpenoids, and alcohols are likely to oxidize to their corresponding aldehydes or ketones.^34^ Therefore, the O_o_[H] compositions observed after the reactions correspond to a combination of oxidation products and nonreactive molecules. Thus, while the TL and HS fractions share a similar VK compositional space, they are composed of chemical compounds with different hydroxyl profiles: carboxylic acids, long chains, and waxes within the TL fraction and sterols––terpenoid-like chemicals in the HS fraction.

A line with slope −2 and intercept at (0,0), also known as “hydration line”, assists in discerning significant differences among the WS, LMWL, and HMWL fractions. Although the compositional space of the raw samples seems similar prior to reaction, distinct characteristics become evident postreaction. Specifically, the O_o_[H] molecular assignments of the WS, LMWL and HMWL fractions exhibit a shift toward lower *H*/*C* values, as indicated by molecular compositions lying below the hydration line. Lower *H*/*C* values can result from the loss of two hydrogen atoms through the oxidation of primary or secondary alcohols.^66^ For instance, consider benzyl alcohol (C_7_H_8_O), which undergoes oxidation to form benzaldehyde (C_7_H_6_O). This shift then indicates the formation of ketones from primary alcohols and aldehydes from secondary alcohols, a reaction with >97% efficiency in DMSO-Ac_2_O solutions.^34^ As shown in Figure 3, the WS fraction exhibited the most significant shift toward lower H/C values after reactions in DMSO-Ac_2_O. Consequently, compared to the water-insoluble material, the WS fraction consists of molecules containing primary and secondary alcohols, for example, sorbitol and levoglucosan, among others. More details on this are presented in the following section.

The O_o_S_s_[H] classes detected in WS and HMWL also showed a displacement toward lower H/C values, indicating a combined reaction involving MTM attachments and oxidations. Given the high unsaturation of these molecules, it is suspected that MTM attaches to the available ortho-, para-, or meta-positions in a benzene ring. It is noteworthy that such a shift toward a low H/C value was observed to a lesser extent in the LMWL fraction. Additionally, in comparison to the HMWL fraction, a higher number of O_o_S_s_[H] heteroatomic classes with lower oxygen and sulfur contents were observed in the LMWL fraction. This indicates a lower availability for MTM attachments in compositions with a higher oxygen content within the LMWL fraction and, consequently, a generally lower content of phenolic compositions. In summary, in contrast to the LMWL, the HMWL is believed to be composed of a combination of a high number of phenolic compositions and primary–secondary alcohols prone to oxidation in DMSO-Ac_2_O.

Until now, our discussions have described the possible hydroxyl distribution of each bio-oil fraction. However, the traditional graphical interpretation lacks meaningful and quantifiable properties from the FT-ICR MS data. The following section presents a suggested alternative for FT-ICR data interpretation.

### 3.2 k-Means Clustering Analysis for Bio-Oil Data Interpretation

#### 3.2.1 Proof-of-Concept: Standard Chemical Compounds

Cluster analysis is an unsupervised machine learning method, used to identify potential patterns and groupings in unlabeled data sets.^40,42^ The goal is to group unlabeled data so that the similarities of the objects in the same group (cluster) are higher when compared to data objects in other clusters.^40^ Our initial objective is to utilize k-means clustering on a set of chemicals reported in the bio-oil literature (see Table S1) to verify if a sensible molecular classification can be achieved with an unsupervised method.

k-Means clustering analysis begins with the user defining the number of clusters, k. Subsequently, the algorithm computes the sum-of-squared distances within the cluster (within sum of squares). This process iterates until the within sum of squares for each cluster is minimized, effectively ensuring that each data point is assigned to the nearest centroid.^40,42,60^ The analysis often includes the consideration of between-cluster variations. The sum of squares measures the distance between clusters, offering insights into their distinctiveness. Ideally, an optimal number of clusters exhibits a small within sum of squares and a large between sum of squares. Determining the optimal number of clusters is a crucial step. Here, the elbow method was used to find the optimal number of clusters. In an elbow analysis, within the sum of squares at different k-clusters plotted, the location of the bend (elbow) in the plot is considered an indicator of an appropriate number of clusters. We have additionally incorporated a criterion where the ratio of the between-cluster sum of squares (*between*_*SS*) to the total within-cluster sum of squares (*total*_*SS*) exceeds 80%, which serves as a threshold for identifying effective clustering.

**Figure 4:**
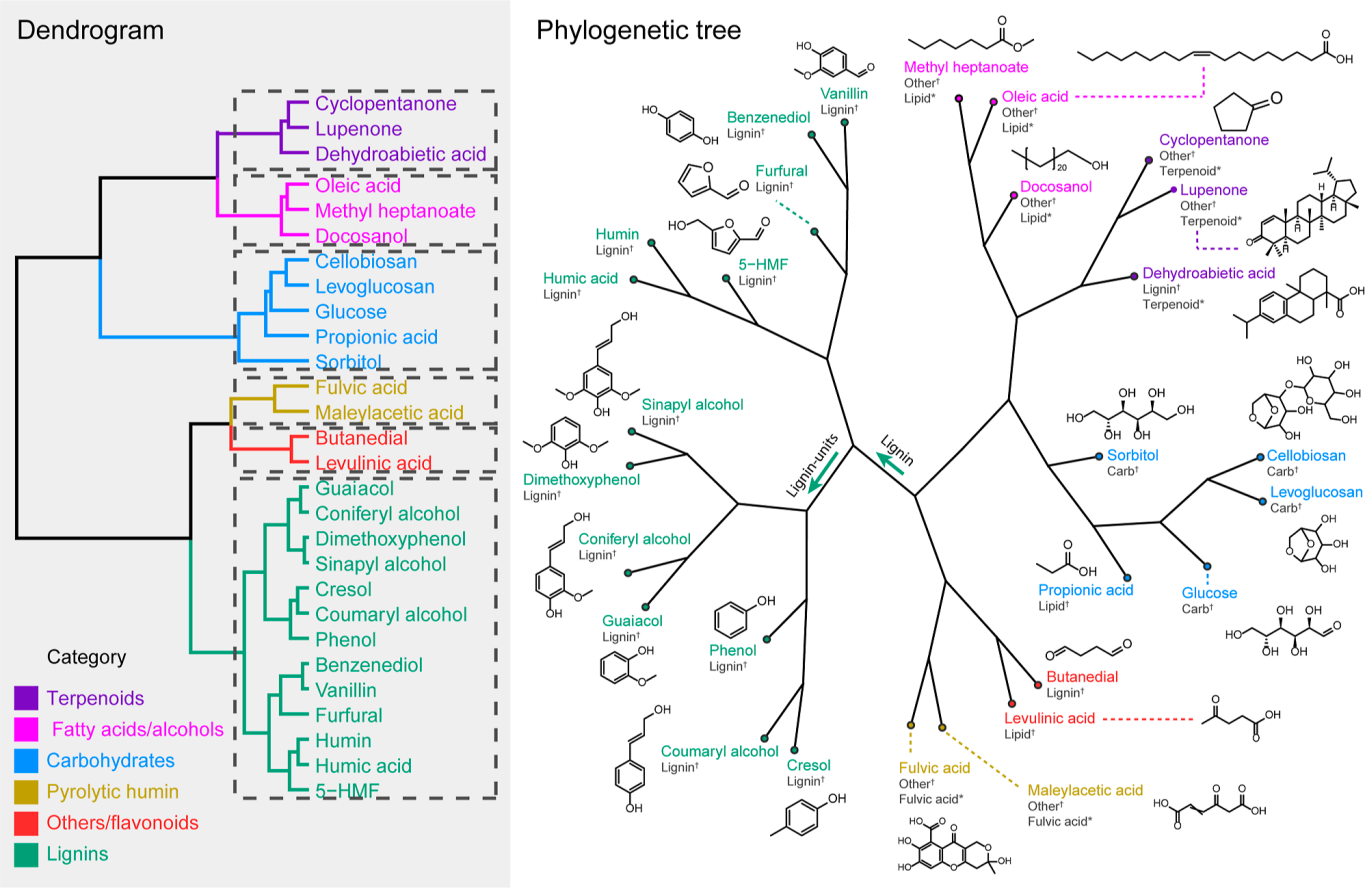
Dendrogram and k-means tree obtained by k-means cluster analysis of standard bio-oil’s molecules. Molecular classification, as suggested in the literature, was included for each chemical: † Hockaday et al.,^35^ * Brockman et al.^38^

In this work, the intracluster and intercluster similarities are based on the Euclidean distance between (*O*/*C*, *H*/*C*) points of the standards. The hierarchical results are represented as both a dendrogram and a k-means tree, as shown in Figure 4. A summary of the statistical results can be found in the Supporting Information.

As shown in Figure 4, k-means analysis indicates that the bio-oil standards can be classified into six clusters. For instance, propyl phenol units, namely, *p*-coumaryl, coniferyl, and sinapyl alcohol and phenolic aldehydes such as vanillin can be found clustered in a single group, whereas long-chain fatty acids and alcohols were clustered together in a separate group. Therefore, our results indicate that a meaningful chemical characteristic can be identified in each cluster. Based on the structural characteristics of each cluster, we have classified the chemicals as follows: terpenoids, long-chain fatty acids/alcohols, carbohydrates, py rolytichumin, others/flavonoids, and pyrolytic lignin compounds. Notably, this classification is consistent with previous literature.^35,38^ However, the cluster analysis distinctly segregates, for example, butanedial (*O*/*C* = 0.5, *H*/*C* = 1.5) into a separate cluster from pyrolytic lignin-like chemicals, despite its composition falling within the typical range for lignin classification (*O*/*C* = 0.1–0.67, *H*/*C* = 1.5–0.7).^35^

Additionally, Figure 4 illustrates the division of pyrolytic lignin-like compounds into two primary groups. One group comprises primary pyrolytic lignin-building units, including *p*-coumaryl, coniferyl, and sinapyl alcohols, which structurally vary in the number of methoxy groups on the benzene ring. These chemicals are linked to an internal branch of the pyrolytic lignin structure (termed the pyrolytic lignin unit clade). Vanillin, benzenediol, humin-like compounds, and others formed a second major group within pyrolytic lignin-type compounds.

A closer examination of the branch grouping within the lignin unit clade reveals that compounds with greater structural similarities, such as coumaryl alcohol and cresol or guaiacol and coniferyl alcohol, are nested within each other, thereby forming their own clades within the pyrolytic lignin units. This observation suggests that the k-means clustering analysis of the compositional space defined by (*O*/*C*, *H*/*C*) can effectively identify groups of molecules with similar structural characteristics.

In contrast to VK diagrams (Figure S1), employing clustering analysis and k-means trees for visualization provides a clear and intuitive approach for extracting and interpreting clustering patterns within the data. It is worth noting that the k-means analysis conducted in this study utilizes the same compositional space information as that of the VK diagram. However, it distinguishes itself by not only enabling the measurement of data point closeness (similarities) but also facilitating the extraction of the number of points clustered within the same centroid.

#### 3.2.2 Clustering Analysis of Bio-Oil Fractions

**Figure 5:**
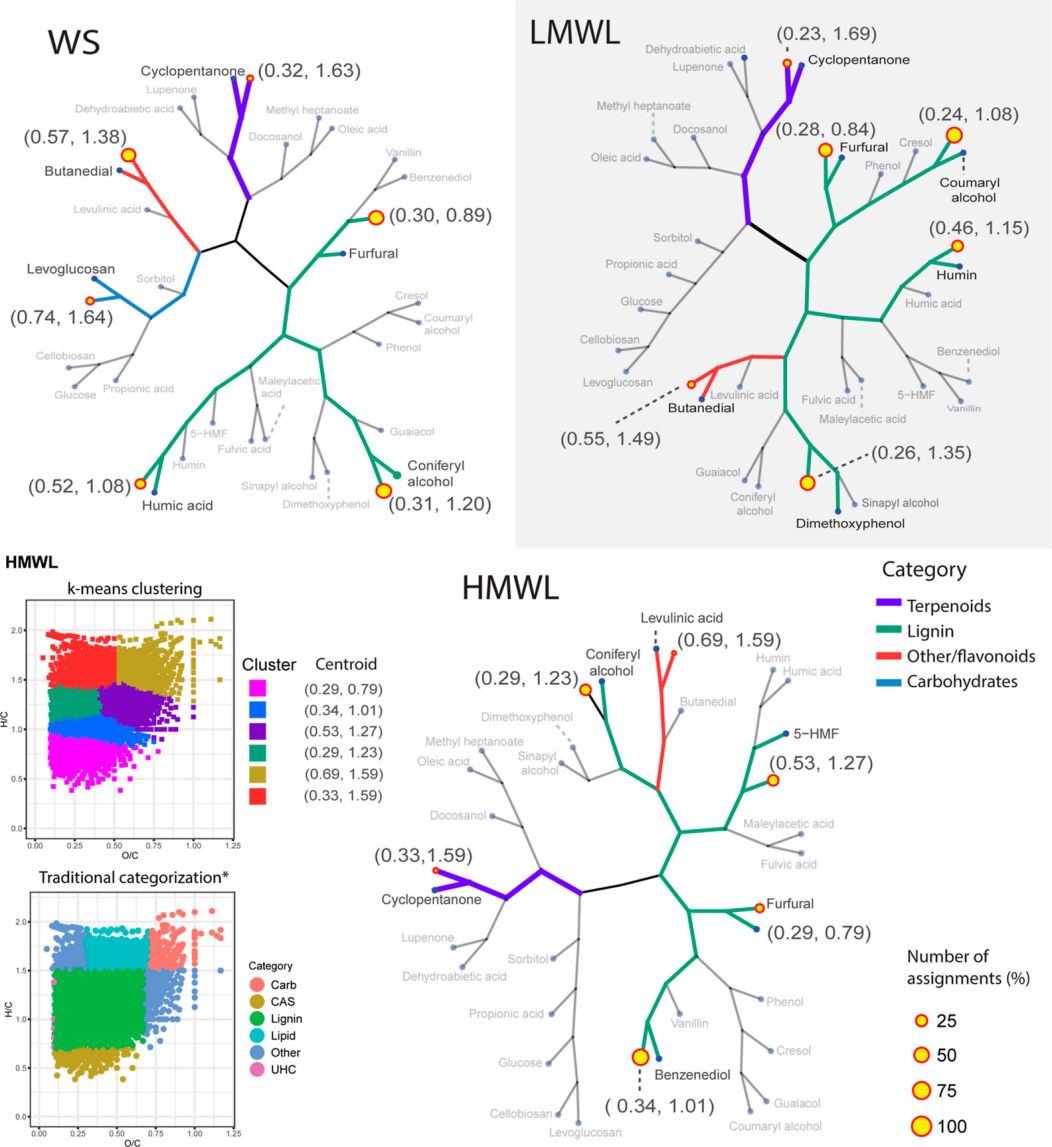
Hierarchical clustering diagrams, represented as k-means trees, illustrating the oxygenated molecular compositions of WS, LMWL, and HMWL fractions of the bio-oil (green branches represent pyrolytic lignin-like, purple––terpenoids, red––flavonoids, and blue––carbohydrates). Bottom-left: VK diagrams comparing oxygenated compounds’ categorization by *Hockaday et al.^35^ (left), where CAS stands for condensed aromatic structures and UHC represents unsaturated hydrocarbons and the categorization performed by k-means clustering (unsupervised machine learning). Centroids correspond to (*O*/*C*, *H*/*C*) coordinates.

In line with the analysis performed on standard chemicals, we utilized k-means clustering of the (*O*/*C*, *H*/*C*) compositional space to assess the potential clustering of the molecular compositions within the bio-oil samples. An example of this procedure is illustrated at the bottom left of Figure 5. Furthermore, we combined the centroids obtained from the bio-oil samples with the (*O*/*C*, *H*/*C*) values of the chemical standards outlined in Table S1. Subsequently, hierarchical clustering was applied with the objective of elucidating possible structural attributes of the molecules within each cluster. Thus, a possible structure characteristic is associated with each bio-oil cluster based on the Euclidean distance to the standard chemicals from Table S1. These results are illustrated in k-means trees, as shown in Figure 5 (refer also to Figure S2 and Table S4 for more details).

Our results reveal that the compositional space of the bio-oil’s fractions can be grouped in 5–6 clusters with a *between*_*SS*/*total*_*SS* > 78.5, indicating a high robustness of the clustering analysis. In comparison to other categorization methods, the shapes of the clusters are irregular and sample-dependent (see the related example in Figure 5). Thus, k-means clustering can be inherent to the unique molecular compositional space in each sample.

**Figure 6:**
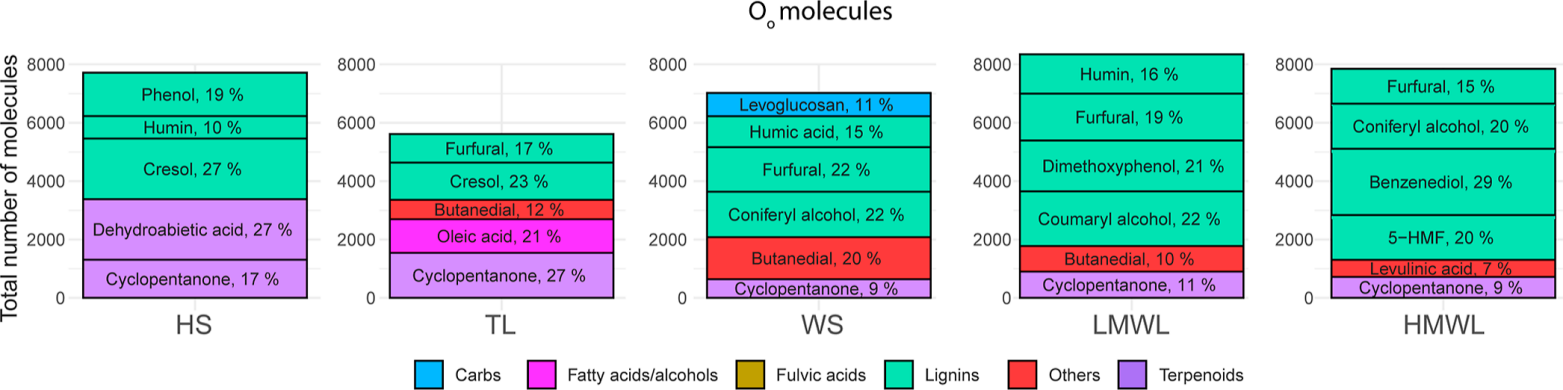
Summary of the percentage of elemental molecular formulas by the cluster of the bio-oil fractions. Molecule names in each bar represent the closest neighbor to each cluster when they were hierarchically compared to the chemical space of bio-oil’s standards from the literature.

Hierarchical analysis was performed over the combined centroids from the FT-ICR data, and the chemical standards allowed us to identify the closest neighbor for each cluster, which in turn can be used to infer the possible structure characteristics. Moreover, the k-means analysis computes centroids along with the number of molecules per cluster for each sample. A summary of these results is presented in Figure 6.

Our results reveal that the molecular formulas detected in the HS and TL samples predominantly clustered into two major families. One family corresponds to terpenoids, while the other corresponds to small pyrolytic lignin-type materials (phenols and cresols). Within the HS fraction, molecular formulas in clusters with the nearest neighbor to dehydroabietic acid and cyclopentanone accounted for 44%. Extractives such as terpenoids, characterized by a low oxygen content, tend to segregate from the polar compositions in the bio-oils in nonpolar solvents. Therefore, our results are consistent with the earlier findings by Oasmaa et al.^44^ Some of these extractives were subsequently separated from the water-soluble fraction, forming an insoluble top layer (TL fraction).

Our cluster analysis indicates that 21% of TL compounds could be closely associated with oleic acid and 27% with cyclopentanone-like chemicals. The TL molecular compositions were, overall, the least reactive in DMSO-Ac_2_O, and therefore these molecules are less likely to contain hydroxyl groups. Therefore, in alignment with our previous discussion, these molecular compositions might correspond to oleic acid material, long-chain methyl esters, resin acids such as abietic acid, and terpenoids such as isopimaric acid. These molecules collectively constitute approximately 48% of the detected compositions and are predominantly located within a zone referred to in the literature as ‘zone 1′ 1.5 ≤ *H*/*C* ≤ 2, 0 < *O*/*C* ≤ 0.3).^21,66^ Therefore, this fraction contains molecules with a higher energy density and elemental molecular formulas similar to those of fossil fuels. Furthermore, the TL fraction also exhibits fewer MTM (methylthiomethyl) attachments after DMSO-Ac_2_O reactions, indicating the presence of a nonreactive isomer of cresol, such as benzyl alcohol, within this sample. This cluster of molecules accounted for 23% of the total molecules detected in this fraction.

In contrast to the TL fraction, the HS fraction was highly reactive in the DMSO-Ac_2_O reactions. This indicates the presence of more hydroxyl groups in this fraction. Previous findings by Palacio Lozano et al.^34^ suggest that hindered phenolic compositions have a reaction yield of 97% in DMSO-Ac_2_O. Thus, the high reactivity of the HS sample in DMSO-Ac_2_O mixtures may be attributed to the abundant availability of ortho-, para-, and meta-positions in the benzene ring of phenols and cresols within the HS sample. Unlike the TL fraction, clusters with a close Euclidean distance to cyclopentanone and dehydroabietic acid may correspond to terpenoids containing hydroxyl groups, such as eugenol, thymol, betulinol, and sitosterol, among others. Some of these compounds have been previously identified in studies conducted by Oasmaa et al.^43,44^ Terpenoids are natural products with a diverse range of applications.^74^ Further investigations to confirm the presence of such compounds could be pertinent for their potential roles as food preservatives, antioxidant agents, and anti-inflammatory agents. Techniques such as GC-MS or GC × GC MS could be employed to validate the presence of the volatile material in this fraction.

A cluster of molecular compositions close to that of levoglucosan was detected within the WS fraction. Carbohydrates such as levoglucosan are characterized by the presence of hydroxyl groups, imparting hydrophilic properties to the molecules. Previous studies by Stankovikj et al. and Mukarakate et al.^75,76^ have identified molecules with similar structures, thereby aligning with our findings. As discussed previously, a large number of molecules with a low *H*/*C* value and high *O*/*C* value were detected in DMSO-AC_2_O-WS in both O_o_[H] and O_o_S_s_[H] heteroatomic groups. We believe that the main reason for this displacement is the oxidation of carbohydrates and other primary or secondary alcohols within the sample. This oxidation in combination with the attachments of *O*-MTM to the hydroxyl groups could explain the O_o_S_s_[H] heteroatomic classes of O_o_S_s_[H] below the hydration line.

Additionally, three clusters near humic acid, furfural, and coniferyl alcohol were found within this fraction. These results are also consistent with previous literature findings.^43,75^ Notably, a significant number of molecular compositions in the WS fractions were found to be closely located with the coordinates of butanedial. Butanedial has been identified as a major constituent of the aqueous fraction of oak pyrolysis bio-oil using GC × GC MS.^76^ FT-ICR MS analysis, however, enables the detection of molecules with larger carbon numbers that cannot be accessed by GC-MS and GC × GC MS systems.

In both the LMWL and HMWL fractions, the highest number of clusters was observed in areas classified as pyrolytic lignin-like compounds, accounting for 78 and 84%, respectively. A significant disparity between the two fractions’ clustering patterns lies in the prevalence of molecules with a close Euclidean distance to benzenediol and coniferyl alcohol in the HMWL fraction, in contrast with coumaryl alcohol and dimethoxyphenol-like material in the LMWL fraction. From the contrasting results from DMSO-Ac_2_O reactions in the HMWL and LMWL fractions, a greater number of molecules were noted below the hydration line in the HMWL fraction. This observation in combination with cluster analysis leads us to hypothesize that the HMWL fraction contains a higher number of phenylpropane β-aryl ether units, also referred to as β-*O*-4-type oligomeric lignin. These pyrolytic lignin dimers feature primary and secondary hydroxyl groups prone to oxidation in DMSO-Ac_2_O solutions, explaining their distinctive behavior.

### 3.3 Characterization of Aged Bio-Oil Samples

#### 3.3.1 Rheology

**Figure 7:**
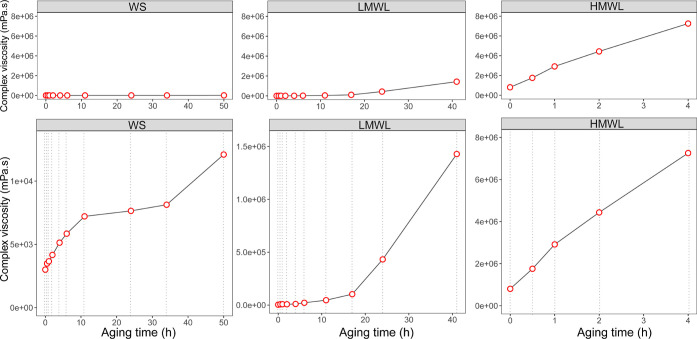
Complex viscosity of the WS, LMWL, and HMWL fractions of a bio-oil. Top: comparison of the complex viscosity using the same *y*-axis scale. Bottom: complex viscosity rescaled per sample.

Fast pyrolysis bio-oils exhibit chemical and thermal instability due to their elevated levels of reactive oxygenated organic compounds.^77^ This instability is often manifested by an increase in viscosity over time, particularly under heating conditions, a phenomenon here referred to as “aging”. The complex viscosity for the WS, LMWL, and HMWL fractions is shown in Figure 7.

Figure 7 illustrates the significant impact of temperature exposure on the rheological characteristics of the bio-oil’s fractions, particularly evident in the HMWL fraction. Exposing the HMWL fraction to elevated temperatures for half an hour resulted in a doubling of the viscosity. Remarkably, within only 4 h, the HMWL was transformed into a highly viscous and sticky material, exhibiting a ninefold increase in viscosity in comparison with the raw material. Conversely, the LWML exhibited a gradual increase in viscosity during the 17 h aging period and only reached a comparable order of magnitude to that of the fresh HMWL sample after 40 h of heating. The aging effects are the least pronounced in the WS fraction. After the sample was heated, minimal changes in viscosity were observed. The viscosity of the WS sample approached that of the fresh LMWL sample after 50 h of accelerated aging. In summary, the impact of aging on viscosity is most pronounced for the HMWL fraction, followed by the LMWL and WS fractions.

#### 3.3.2 Elemental Composition Profile

**Figure 8:**
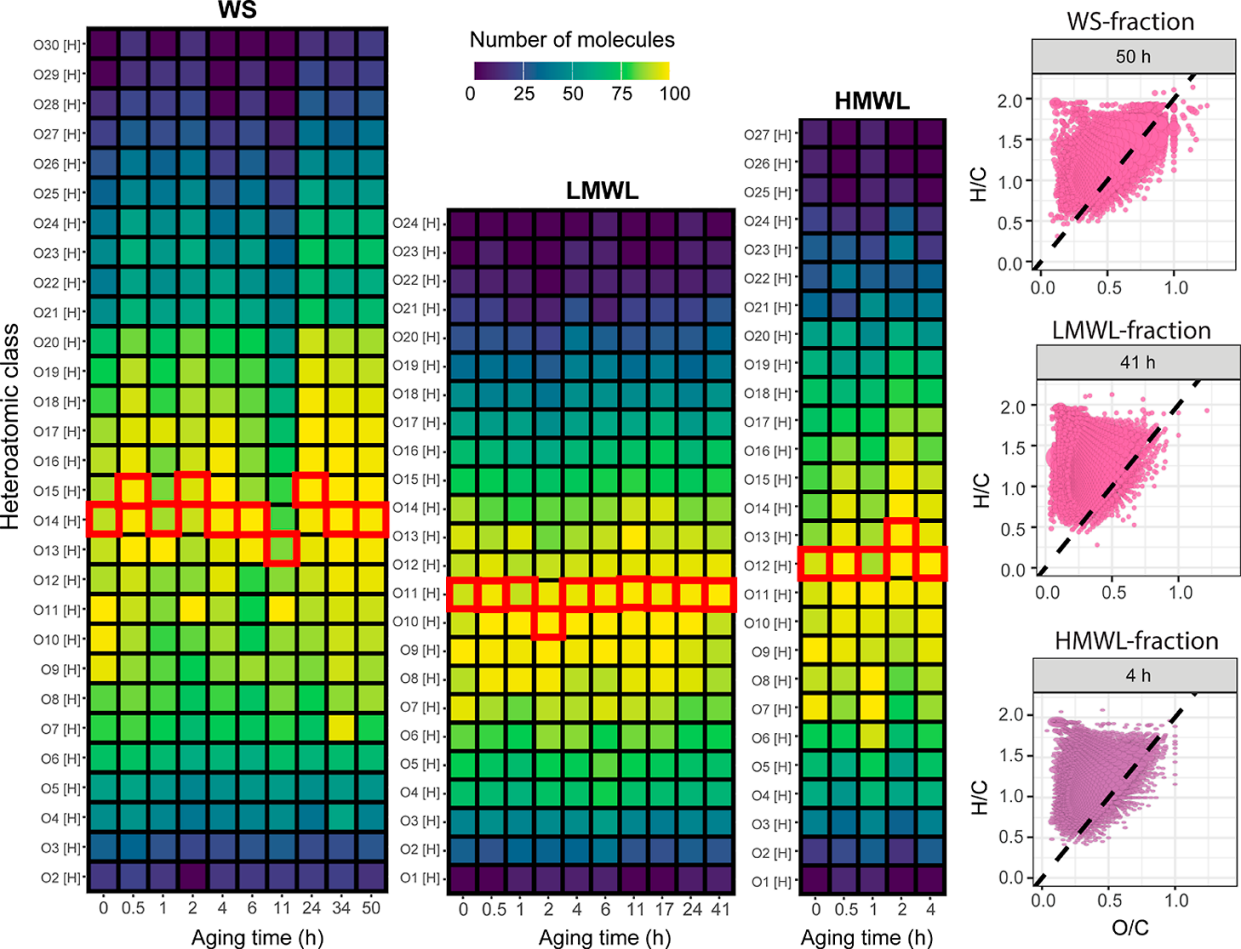
Left: tile plots representing the O_o_[H] heteroatomic class distribution of the accelerated aging experiments of the WS, LMWL, and HMWL fractions of a bio-oil. The red square marks the mean oxygen value. Right: VK diagram of the last aging test point. The black dotted line represents the dehydration line with slope −2.

On average, approximately 9500 elemental compositions were detected in each aging experiment (refer to Table S3 for complete details). Figure 8 presents the heteroatomic class distribution of the oxygenated classes of the WS, LMWL, and HMWL fractions at different aging times. As shown in this figure, the WS aged samples presented the highest oxygen-containing heteroatomic classes compared to both LMWL and HMWL. Conversely, the LMWL samples contain heteroatomic classes with the lowest number of oxygen atoms. Minimal changes in the mean oxygen content were observed throughout the different aging times for each fraction.

The VK diagrams for each fraction at the final aging time are shown on the right-hand side of Figure 8, with additional VK diagrams available in Figures S3–S5 in the Supporting Information. Generally, VK diagrams illustrated a consistent compositional space across aging times, except for the WS fraction at 11 h of aging and the LWML fraction exposed to high temperatures for 1 h, where some molecular compositions were observed below the dehydration line. Similarly, the carbon number, *m*/*z*, and DBE distribution of the full set of molecules detected in each fraction remained comparable before and after the aging experiment (see Figure S6).

#### 3.3.3 Evaluation of Molecular Weight by 21 T FT-ICR MS

The number-average molecular weight and the weight-average molecular weight (Mn and Mw respectively) for WS, LMWL, and HMWL at the two extreme aging points are presented in Table 3. The mass spectra are presented in Figure S7. Ions with the *m*/*z* range between 160 and 1150 and with an average *m*/*z* 499–*m*/*z* 587 were detected by FT-ICR MS in negative-ion mode. Interestingly, a similar mean molecular weight was found in between the so-called “LMWL” fraction and the “HMWL” fraction (*M*_n_ ∼ *m*/*z* 504). A fractionation study performed in bio-oils by Garcia-Perez et al.^78^ showed very contrasting results to the ones observed by FT-ICR MS. In their study, Garcia-Perez conducted multiple fractionation steps, starting with the extraction of toluene-soluble fractions. This was followed by a similar separation process for water-soluble, water-insoluble/CH_2_Cl_2_-soluble, and water-/CH_2_Cl_2_-insoluble fractions. Gel permeation chromatography (GPC) was utilized to analyze the molar mass distribution. Contrary to our findings, Garcia-Perez et al.^78^ estimated a molar mass distribution between 538 and 1000 g/mol for the water-insoluble/CH_2_Cl_2_-soluble fraction (comparable to the LMWL fraction in this article), while the water-/CH_2_Cl_2_-insoluble fraction (analogous to the HMWL fraction) was found to have a mass distribution between 1475 and 1967 g/mol.

**Table 3:** Number-Average and Weight-Average Molecular Weights of the Fractions WS, LMWL, and HMWL at 0 and the Highest Oxidation Points

sample id	Mn	Mw
HMWL 0h	516.4452	587.0907
HMWL 4h	499.9043	571.3782
LMWL 0h	491.6011	553.4821
LMWL 41h	477.9793	548.6292
WS 0h	487.2953	548.7878
WS 50h	506.2617	569.8406

Significant discrepancies in molecular mass distribution assessed by GPC and FT-ICR MS (or mass spectrometry methods in general) have been previously documented in asphaltene analysis^79^ and recently reviewed by Harman-Ware and Ferrell III for bio-oil analysis.^80^ The molecular weight distribution is an important metric of bio-oil characterization; however, it is not a trivial subject. We hypothesize that, similar to the findings in asphaltene studies, the increased heteroatomic content, as noticed by the high oxygen content for the HMWL fraction in Figure 5, results in stronger intermolecular interactions, influencing the viscosity of the sample and the molecular weight observed in GPC analysis. It is also possible that aggregates are present at low concentrations, making their detection difficult. Further studies are needed to confirm these hypotheses.

As shown in this section, there are few apparent differences in the overall elemental compositions of the bio-oil fractions that explain their varying viscosity behavior during aging experiments. In the subsequent section, we will use k-means clustering analysis to provide a more insightful interpretation and understanding of bio-oil aging.

#### 3.3.4 Clustering Analysis of Bio-Oil’s Fractions after Aging Experiments

**Figure 9:**
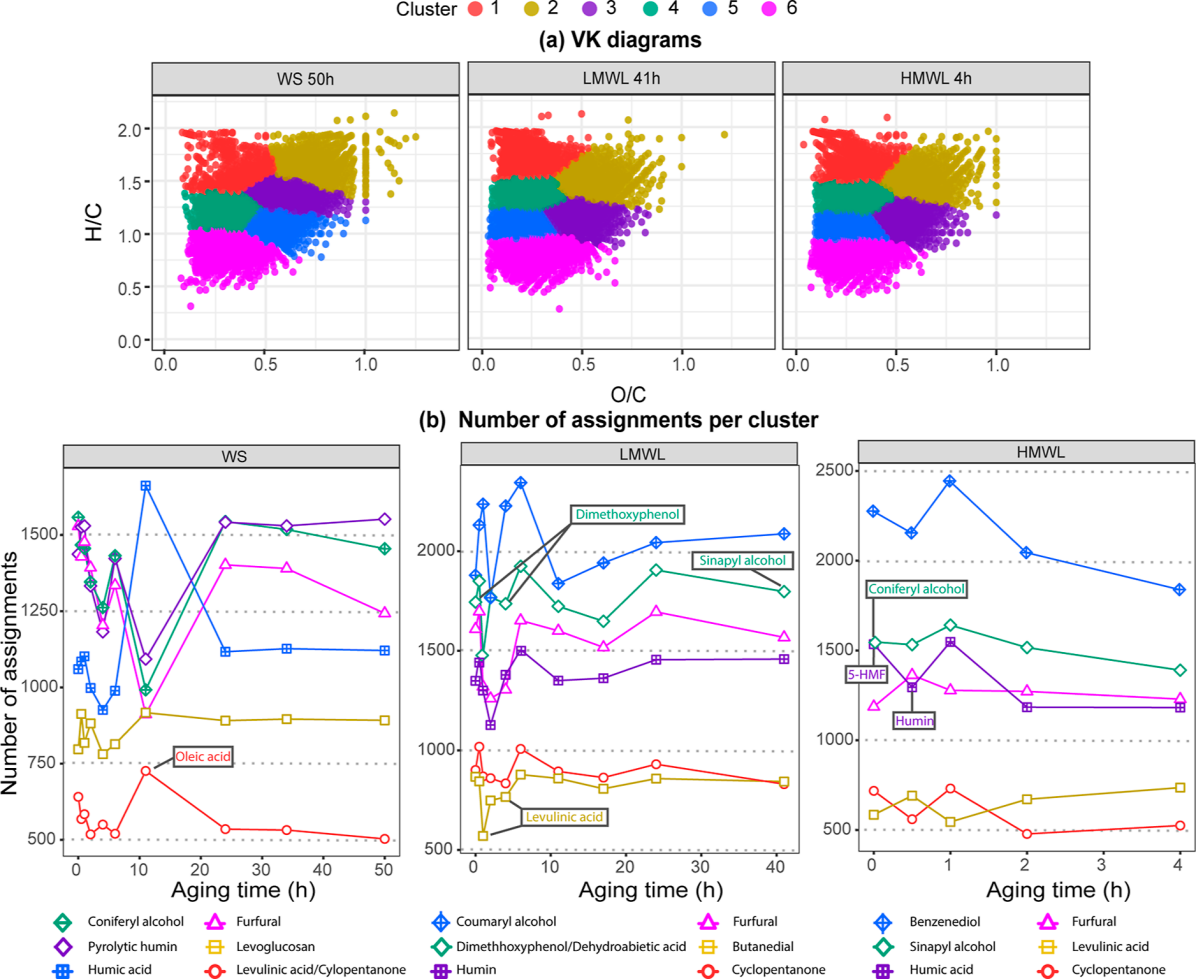
Number of molecules in the Oo[H] heteroatomic class per cluster at different aging times. The nearest neighbor for each fraction is shown below each fraction.

The FT-ICR MS data of the aging fractions of WS, LMWL, and HMWL were also subject to k-means clustering analysis. The results are summarized in Figure 9 and Table S5. Additional information including VK diagrams, and *C*, *O*, and *m*/*z* distributions, can be found in Figures S8–S12. It is worth noting that, in contrast to the overall elemental molecular comparison shown in Figure S7, clear changes in DBE, carbon number, and oxygen distributions can be observed in each cluster when k-means cluster analysis is performed.

As shown in Figure 9, the WS fraction presented a different grouping pattern to the one observed for the LMWL and HMWL fractions. Note for instance the contrast of clusters 2, 3, and 5 at high *O*/*C* values in WS in comparison to only two clusters (2 and 3) at those *O*/*C* values in the water-insoluble material. This demonstrates how k-means clustering identifies patterns within the data tailored to the detected molecular composition of the sample, in contrast to using predetermined *H*/*C* and *O*/*C* value ranges for classifying and semiquantifying data into families. These clusters correspond to molecules with a Euclidean distance close to levoglucosan, pyrolytic humins, and humic acid materials (clusters 2, 3, and 5, respectively). Pyrolytic humin material in bio-oils was proposed by Stankovikj et al. in 2016 and corresponds to highly dehydrated oligomeric compounds derived from the decomposition of cellulose and hemicellulose.^75,81,82^

The high *O*/*C* ratio observed in these clusters is a consequence of the predominance of oxygen-rich moieties with the corresponding reduced number of rings and double bonds (refer to Figures S8–S10 for further details).

Some structural characteristics can be inferred from these clusters as follows. Cluster 2, for instance, consisting of an average of 850 molecules across aging time points, is characterized by an average DBE of 5 and an average oxygen count (O_mean_) of 15. Consequently, the presence of phenolic units, each one contributing with a DBE of 4, is unlikely to be present within this cluster. Instead, polyols, such as sugar alcohols, exemplified by levoglucosan, better typify the molecular moieties of this cluster. Analogous reasoning can be applied to molecules situated within clusters 3 and 5. Molecules in these clusters might correspond to the so-called “pyrolytic humin”, the highly dehydrated oligomeric compounds derived from cellulose and hemicellulose.^81^ The structure of pyrolytic humin has been hypothesized to consist of molecules with both aromatic- and carbohydrate-like features.^75^ Such structures could explain the increased DBE value of the molecules in clusters 3 and 5 within the WS fraction. However, note that the molecules in clusters 3 and 5 generally showed a significant shift toward lower DBE values compared to the same molecules detected in the LMWL and HMWL fractions. This suggests that pyrolytic humin-like moieties are less likely in the water-insoluble fractions (which, in contrast, contain pyrolytic lignin).

As highlighted in the preceding sections, the presence of primary and secondary alcohols in this fraction was evidenced by the combination of *O*-MTM attachments and the oxidation of primary and secondary alcohols in DMSO-Ac_2_O mixtures, indicating a notable content of primary and secondary hydroxyl groups. The WS fraction differs from the LMWL and HMWL fractions by having a significantly higher content of oxygen in carbohydrate-like structures. Furthermore, WS samples showed minimal changes in complex viscosity during the aging periods of up to 50 h of heating. This suggests that a high oxygen content does not necessarily result in increased viscosity, underscoring the pivotal role of oxygen functionality in bio-oil aging.

Levoglucosan, derived from cellulose degradation, can undergo transformation into furfural during pyrolysis via mechanisms involving ring scission and recyclization.^83^ Thus, it is expected that all samples exhibited an agglomeration of molecules close to the furfural-like material. As illustrated in Figure 9, this cluster of molecules was the most prominent in the WS fraction, followed by the LMWL and HMWL fractions.

Unlike the WS fraction, both LMWL and HMWL exhibit two distinct clusters situated within the compositional space corresponding to pyrolytic lignin-building unit moieties (clusters 4 and 5, pyrolytic lignins), which collectively contribute the highest number of molecular assignments for these fractions. It is interesting to note that the compositions located in cluster 5 have molecules distributed toward higher *m*/*z* values (see Figure S9). This pyrolytic lignin-type molecule is a conformation of sequence of monomeric units covering a mass range of about *m*/*z* 250–1000 with an average molecular mass of *m*/*z* 740.

A discernible distribution of pyrolytic lignin-building units of cluster 5 was observed between the LMWL and HMWL. For instance, while cluster 5 in the LMWL corresponds to coumaryl alcohol-like structures, in the HMWL, it corresponds to benzenediol. Hydroxybenzenes such as catechol, resorcinol, and hydroquinone are organic compounds featuring two hydroxyl groups attached to a benzene ring. In contrast, coumaryl alcohol features an allylic alcohol moiety connected to the para position of the phenolic group. This observation suggests that the pyrolytic HMWL contains either a higher number of –OH attachments to a phenyl propane unit or a greater abundance of phenolic units in comparison with the molecules observed in the LMWL. Phenolic compounds, the known culprits in accelerating the aging rate of bio-oils,^11,12^ likely contribute to the significantly elevated viscosity observed in the HMWL.

Molecules found in cluster 4 in the HMWL, also in the area corresponding to lignin-building units, were observed close to coniferyl and sinapyl alcohol units, indicating a higher content of methoxy groups within this sample. Interestingly, at 41 h of accelerated aging, cluster 4 in the LMWL similarly exhibited molecules clustering near a sinapyl alcohol-like structure. Thus, this indicates an increase of methoxy groups of the LMWL molecules after aging, probably as a consequence of polymerization reactions.

An interesting discussion of lignin interunit bondings presented by Crestini et al.^84^ indicates that a linear lignin polymeric unit linked by β-*O*-4 aryl ether and a phenylcoumaran (β-5′) interunit linkage yields a single phenolic–OH unit per polymer chain, whereas β–β and β-1 bonding produce a dilignol bearing two terminal phenolic groups. Subsequent studies employing the MS/MS analysis of aged bio-oil fractions with ultrahigh-resolution mass spectrometry could offer deeper insights into the types of lignin interunit likely present within these samples, particularly focusing on molecular compositions detected within each different k-mean cluster.

Thousands of individual peaks can be found in a single high-resolution mass spectrum, leading to difficulty when performing targeted MS/MS analysis. Clustering algorithms such as k-means can aid in identifying “areas of interest” for isolation and consequent fragmentation. However, such experiments are currently beyond the scope of this paper.

## 4 Conclusions

This paper describes how a combination of a targeted derivatization method for sample pretreatment before FT-ICR MS analysis and unsupervised k-means clustering analysis can be used to interpret the structural characteristics of bio-oil fractions and the effect of hydroxyl functionality on aging stability. The following conclusions can be drawn.

Dilution of the samples in DMSO-Ac_2_O solutions prior to FT-ICR MS analysis helped to identify the presence of a variety of hydroxy groups within the fractions. The HS fraction exhibited the highest abundance of primary and secondary alcohols, which likely undergo combined oxidation and *O*-MTM attachment reactions. Conversely, the TL fraction displayed a lower reactivity in DMSO-Ac_2_O, indicating the presence of reduced number of hydroxyl groups. VK diagrams of the WS and HMWL fractions revealed numerous compositions lying below a hydration line, suggesting oxidation of primary and secondary alcohols. Furthermore, the abundance of the O_o_S_s_[H] compositions in the WS, LMWL, and HMWL fractions suggests the presence of phenolic groups with MTM chains attached to the available para-, ortho-, or meta-positions in the benzene rings or the attachment of an *O*-MTM to sterically hindered alcohols.

Accelerated aging experiments were conducted on the WS, LMWL, and HMWL fractions. Rheology experiments revealed that the WS fraction exhibited minimal changes in viscosity even after exposure to temperatures of up to 80 °C for up to 50 h. Similarly, the LMWL fraction showed stable viscosity levels during the initial 10 h of heating, but prolonged exposure resulted in increased viscosity. In contrast, the HMWL fraction exhibited remarkably high viscosity, undergoing rapid transformation into a highly viscous and sticky material within just 4 h of aging. This characteristic is potentially analogous to asphaltene-like components found in crude oil samples, indicating that HMWL may present the greatest challenges during bio-oil upgrading processes. Our results suggest that removing the water-/CH_2_Cl_2_-insoluble material (HMWL) from bio-oils can significantly reduce aging effects during storage. This material could be used to produce resins, such as wood adhesives. Future studies on this topic are recommended.

k-Means clustering analysis of the compositional space defined by (*O*/*C*, *H*/*C*) can effectively identify groups of molecules with similar structural characteristics. Unlike other methods documented in the literature, k-means clustering can be tailored to the unique compositional space of each sample, thereby offering semiquantification of compositions within each cluster.

k-Means clustering analysis of the bio-oil fractions allows us to infer possible general structural characteristics of the clusters. Our results indicate that 44% of molecules of the HS fraction were clustered close to a terpenoid-like material, the result that aligns with our observations of the reactions in DMSO-Ac_2_O. Similarly, oleic acid and cyclopentanone-like molecules were observed within the TL fraction, whereas clusters of molecules close to carbohydrates were uniquely observed within the WS fraction.

Finally, k-means cluster analysis revealed that approximately 28% of the molecular compositions in the HMWL fraction are clusters close to benzenediol-like material, indicating a significantly higher number of –*OH* attachments to phenolic units. Additionally, the presence of a second cluster of molecules to sinapyl alcohol suggests polymerization reactions occurring within this material. We speculate that the chemical composition within these clusters likely contributes to the significantly higher viscosity observed in this sample.

Our study reveals that despite significant changes in viscosity, similar molecular compositions persist across varying aging times, highlighting the complexity of bio-oil aging processes. While our investigation did not encompass MS/MS experiments, such analyses hold promise for elucidating structural changes in molecular compositions and their aging products. Additionally, employing chemoselective reactions to target the hydroxyl group under accelerated aging conditions could provide valuable insights into chemical transformations over time. Furthermore, studies that include accelerated aging of standard chemicals combined with chemical derivatization can shed light on the aging mechanisms. These avenues for further exploration offer promising directions for advancing our understanding of bio-oil aging mechanisms.

## Data Availability

The data underlying this study are available in the published article, in its Supporting Information, and openly available in http://wrap.warwick.ac.uk/.

## Supporting Information Available

Statistical data supporting k-means analysis (XLSX)
Cluster analysis of bio-oils, k-means trees, distance to nearest neighbor reported for bio-oil fractions and accelerated aging experiments, complementary VK plots, carbon number, and DBE mean distributions (PDF)
