# Supplementary material for: Assessment of Accelerated Aging Effect of Bio-Oil Fractions Utilizing Ultrahigh-Resolution Mass Spectrometry and k-Means Clustering of van Krevelen Compositional Space
Source: Energy Fuels. 2024 Aug 20;38(17):16473–89. doi: 10.1021/acs.energyfuels.4c02605 (PMC11382156; doi:10.1021/acs.energyfuels.4c02605)
Supplement: Supplementary file 2 — ef4c02605_si_002.pdf [file ef4c02605_si_002.pdf]

Supporting information

Assessment of accelerated aging effect of bio-oil fractions utilizing Ultrahigh Resolution Mass Spectrometry and k-means clustering of van Krevelen compositional space

Diana Catalina Palacio Lozano[*†], Daniel Lester,[¥] JS Town,[¥] Amy M. McKenna[‡,Ȓ] and Martin Wills[†]*

[†]Department of Chemistry, University of Warwick, Coventry, CV4 7AL, UK.

[¥]Polymer Characterisation Research Technology Platform, University of Warwick, Coventry, CV4 7AL, UK.

[‡]National High Magnetic Field Laboratory, Florida State University, 1800 East Paul Dirac Drive, Tallahassee, Florida 32310-4005, United States

[Ȓ] Department of Soil and Crop Sciences, Colorado State University, Fort Collins, CO, 80523, USA

Cluster analysis -bio-oil fractions

Table S 1. Compositional space of standard molecules used for K-means clustering analysis.

H/C	O/C	Molecule
1.11111	0.22222	Coumaryl alcohol

1.2	0.3	Coniferyl alcohol
1.27273	0.36364	Sinapyl alcohol
2.33333	1	Sorbitol
2	1	Glucose
1.6	0.6	Levulinic acid
1	0.5	5-HMF
2	0.25	Methyl heptanoate
1	0.375	Vanillin
1.88889	0.11111	Oleic acid
1.6	0.03333	Lupenone
1.66667	0.83333	Levoglucosan
1.4	0.1	Dehydroabietic acid
2.09091	0.04545	Docosanol
1.83333	0.91667	Cellobiosan
0.8	0.4	Furfural
1	0.16667	Phenol
1.14286	0.28571	Guaicol
1.14286	0.14286	Cresol
1	0.33333	Benzenediol
2	0.66667	Propionic acid
1	0.83333	Maleylacetic acid
1.5	0.5	Butanedial
1.6	0.2	Cyclopentanone
1.1	0.5	Humic acid[1]
1.28	0.76	Fulvic acid[1]
1.17	0.46	Humin
1.2	0.4	Trimethoxybenzaldehyde
1.25	0.375	Dimethoxyphenol

Table S 2. Complex viscosity in mPa.s of the water soluble (WS), low and high molecular weight lignin (LMWL, HMWL respectively).

WS		LMWL		HMWL	
h	Complex Viscocity (mPa.s)	h	Complex Viscocity (mPa.s)	h	Complex Viscocity (mPa.s)
0	3.01E+03	0	3.31E+03	0.5	8.04E+05
0.5	3.49E+03	0.5	6.97E+03	1	1.76E+06
1	3.66E+03	1	7.73E+03	2	2.91E+06
2	4.17E+03	2	7.42E+03	4	4.44E+06
4	5.13E+03	4	1.02E+04		
6	5.85E+03	6	2.16E+04		
11	7.21E+03	11	1.43E+06		
28	7.65E+03	17	1.03E+05		
34	8.13E+03	24	4.32E+05		
50	1.21E+04	41	1.43E+06		

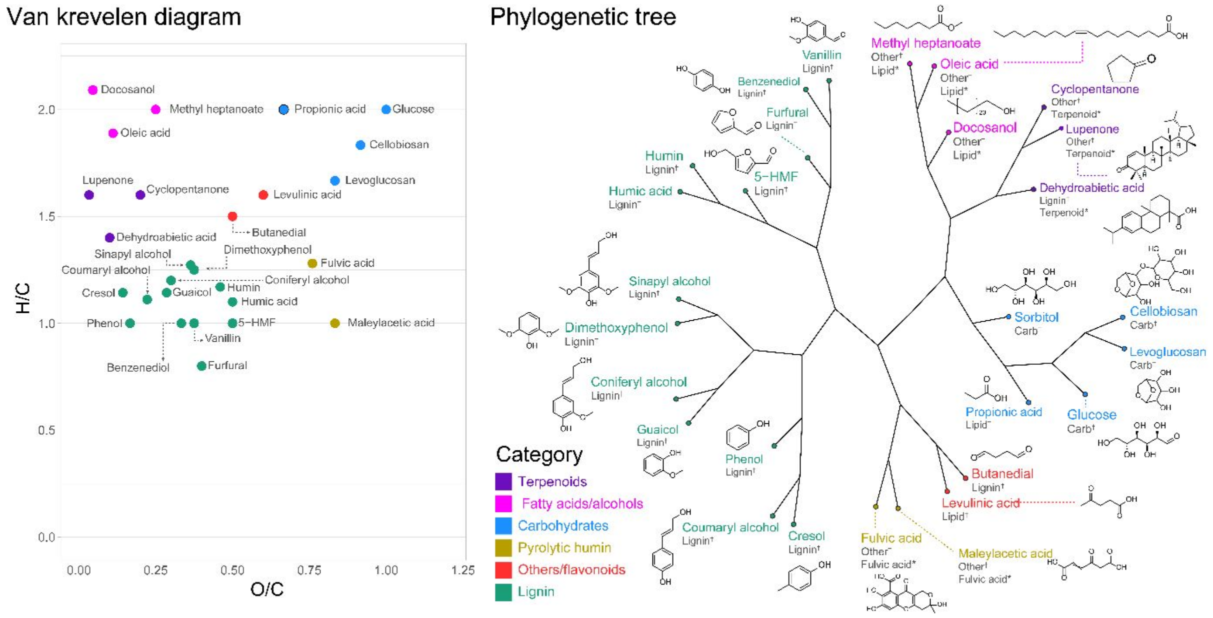

Figure S 1. Left: van Krevelen diagram of bio-oil's standard chemical compounds. Right: k-means tree representing the results obtained by k-means clustering analysis.

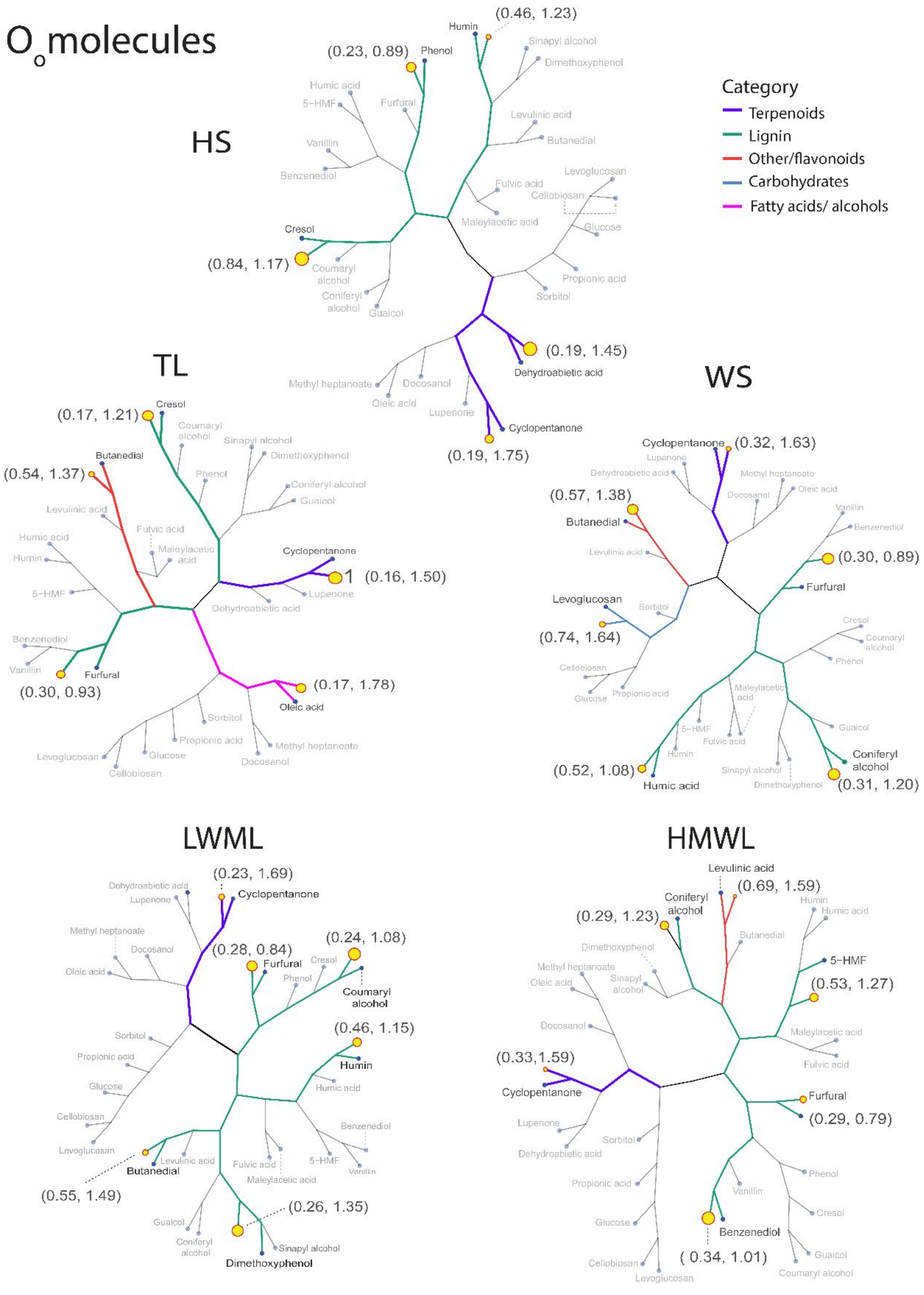

Figure S 2. Clustering analysis of the oxygenated heteroatomic classes of the bio-oil's fractions before reactions in DMSO-Ac$_2$O. Centroids correspond to (O/C, H/C) coordinates.

Accelerated aging

Table S 3. Total number of assignments of WS, LMWL and HMWL bio-oil's fractions across the different aging times.

WS			LMWL			HMWL		
aging (h)	O_o[H]	N_n[H]	aging (h)	O_o[H]	N_n[H]	Aging (h)	O_o[H]	N_n[H]
0	7026	2205	0	8352	1549	0	7856	2878
0.5	6989	1618	0.5	9015	1572	0.5	7601	2631
1	6968	2414	1	8295	2033	1	8202	4157
2	6470	1778	2	7532	1525	2	7170	2474
4	5906	1551	4	8250	1202	4	6903	2025
6	6519	1522	6	9313	1684	NA	NA	NA
11	6774	2648	11	8263	1312	NA	NA	NA
24	7033	1541	17	8138	1601	NA	NA	NA
34	6995	1635	24	8890	1418	NA	NA	NA
50	6767	1321	41	8582	1303	NA	NA	NA

Table S 4. Total number of molecules (N) per cluster of the raw bio-oil's fractions. Results obtained by k-means clustering analysis of FT-ICR MS data.

Raw bio-oil fractions				Cluster centroid		Distance to neighbor
**id**	**Closest neighbor**	**Category**	**N**	**O/C**	**H/C**	$d = \sqrt{(x_2-x_1)^2 + (y_2-y_1)^2}$
HS	cyclopentanone	Terpenoids	1307	0.194	1.748	0.148
HS	Cresol	Lignin	2080	0.184	1.178	0.054
HS	Phenol	Lignin	1488	0.236	0.894	0.127
HS	Humin	Lignin	768	0.459	1.234	0.064
HS	Dehydroabietic acid	Terpenoids	2073	0.191	1.455	0.106
WS	Cyclopentanone	Terpenoids	641	0.320	1.635	0.125
WS	Coniferyl alcohol	Lignin	1558	0.311	1.200	0.011
WS	Humic acid	Lignin	1060	0.526	1.086	0.029
WS	Levoglucosan	Carbs	797	0.736	1.643	0.100
WS	Butanedial	Others	1437	0.570	1.380	0.138
WS	Furfural	Lignin	1529	0.302	0.894	0.135
TL	Cyclopentanone	Terpenoids	1542	0.160	1.502	0.106
TL	Cresol	Lignin	1275	0.175	1.213	0.077
TL	Oleic acid	Terpenoids	1152	0.175	1.786	0.121
TL	Butanedial	Others	665	0.539	1.372	0.134
TL	Furfural	Lignin	978	0.301	0.926	0.160
LMWL	Cyclopentanone	Terpenoids	901	0.228	1.696	0.1
LMWL	Sinapyl alcohol	Lignin	1741	0.260	1.355	0.132
LMWL	Butanedial	Lignin	874	0.549	1.489	0.050
LMWL	Humin	Lignin	1351	0.462	1.156	0.014
LMWL	Coumaryl alcohol	Lignin	1876	0.236	1.088	0.027
LMWL	Furfural	Lignin	1604	0.285	0.844	0.123

HMWL	Cyclopentanone	Terpenoids	718	0.326	1.588	0.127
HMWL	Coniferyl alcohol	Lignin	1544	0.290	1.235	0.036
HMWL	Levulinic acid	Others	583	0.687	1.592	0.087
HMWL	5-HMF	Lignin	1537	0.530	1.268	0.233
HMWL	Benzenediol	Lignin	2272	0.341	1.011	0.014
HMWL	Furfural	Lignin	1194	0.287	0.787	0.114

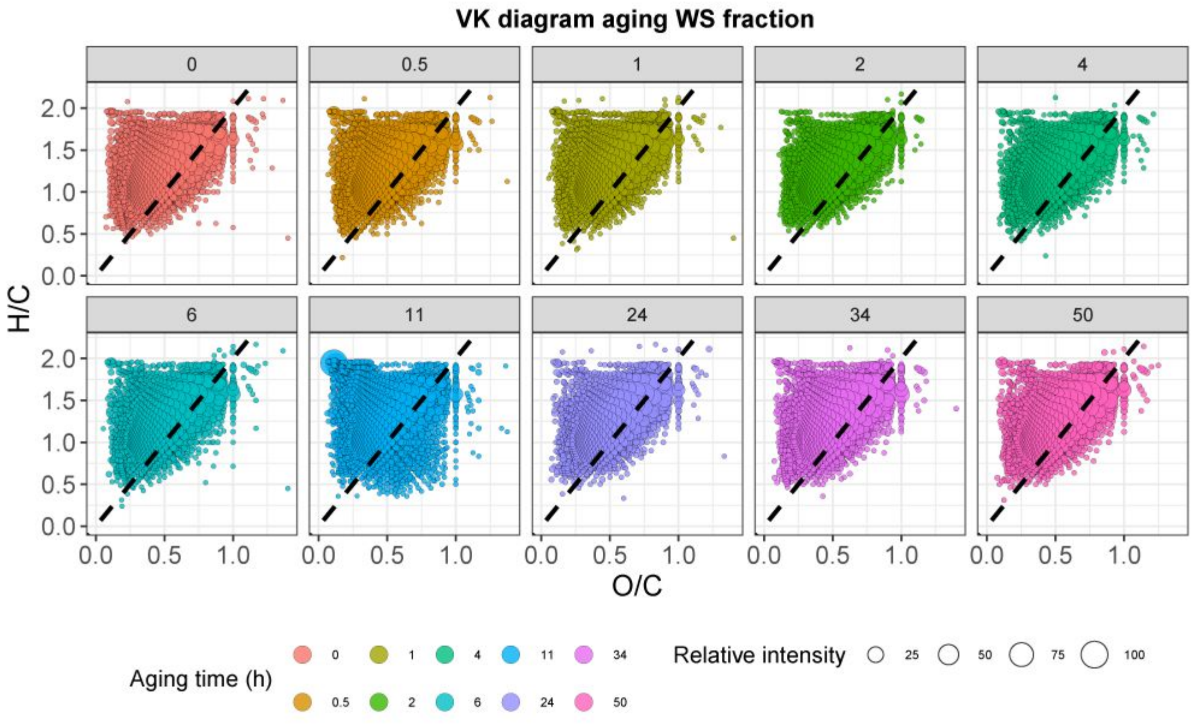

Figure S 3. Van Krevelen diagram of the O_o[H] heteroatomic class for the WS fractions at the different aging collection times. The data was obtained by negative-ion mode ESI FT-ICR MS.

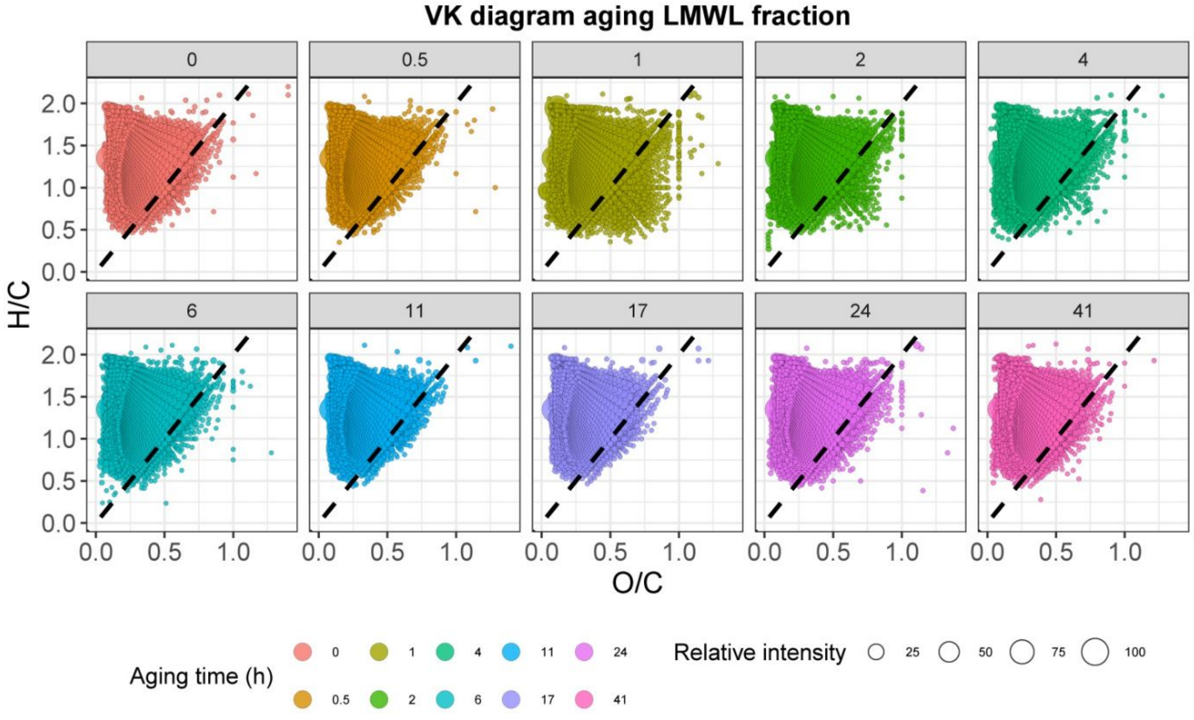

Figure S 4. Van Krevelen diagram of the O_o[H] heteroatomic class for the LMWL fractions at the different aging collection times. The data was obtained by negative-ion mode ESI FT-ICR MS.

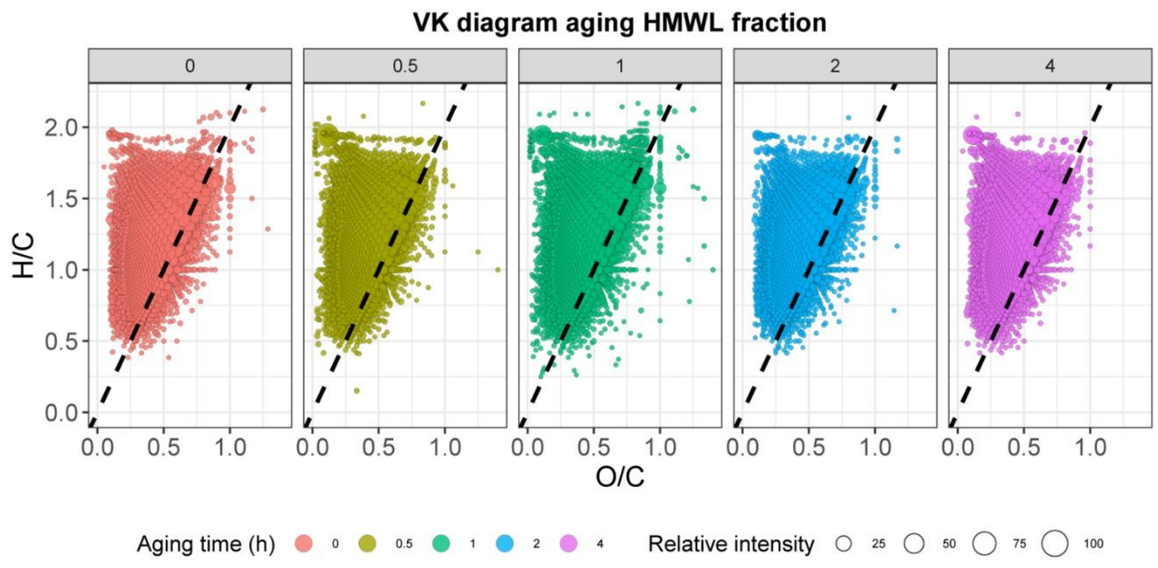

Figure S 5. Van Krevelen diagram of the O_o[H] heteroatomic class for the HMWL fractions at the different aging collection times. The data was obtained by negative-ion mode ESI FT-ICR MS.

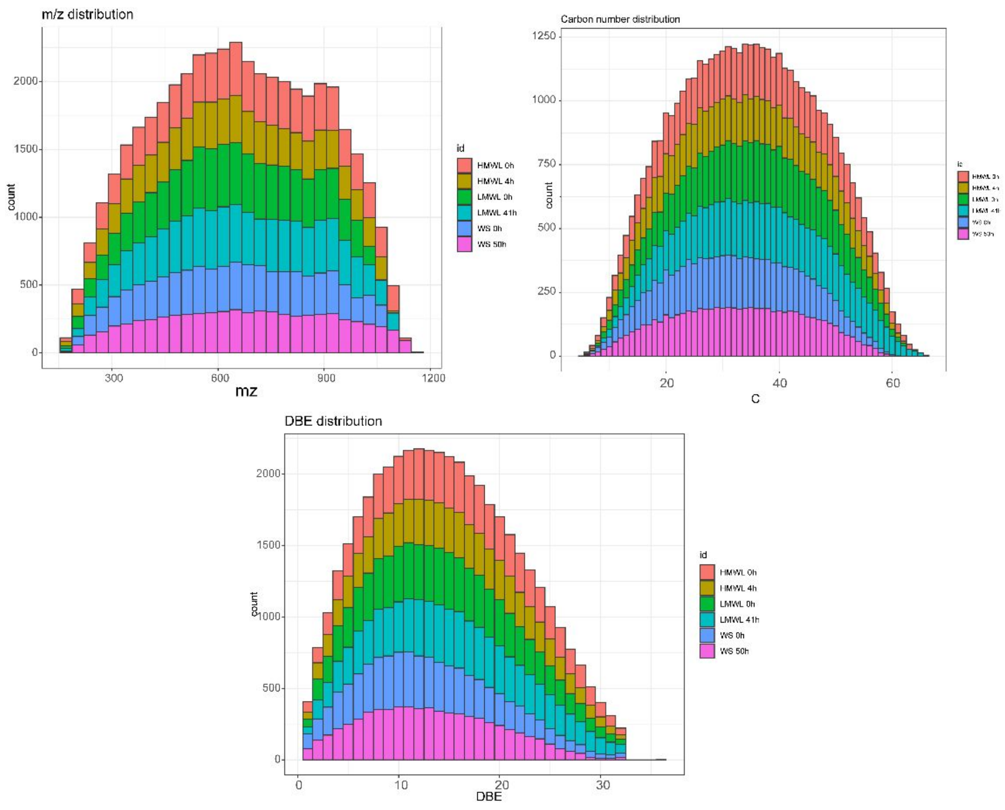

Figure S 6. Carbon number, DBE and m/z distribution of the oxygenated heteroatomic classes of bio-oil fractions at the two extreme aging times.

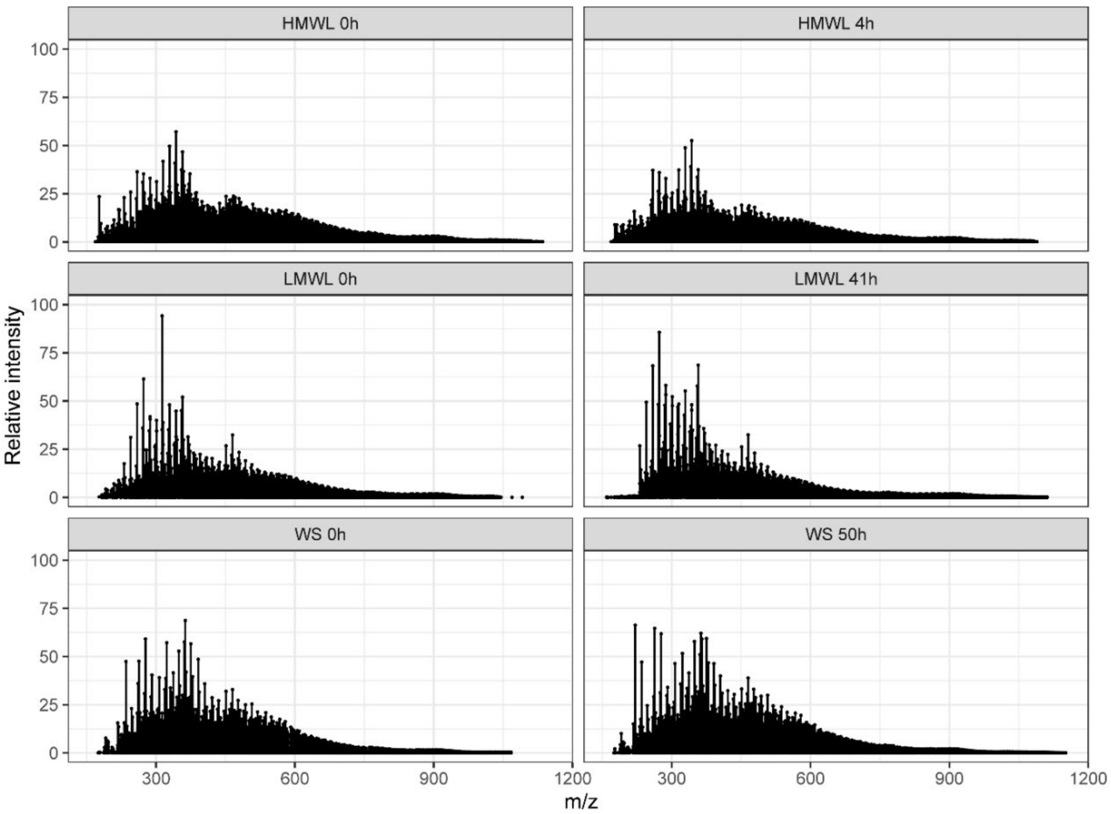

Figure S7. Mass spectra of the fractions WS, LMWL and HMWL without aging 0h and at the highest oxidation point.

Cluster analysis of accelerated aging fractions

Table S 5. K-means clusters of fractions of a bio-oil exposed to different aging times.

id	hours	H.C	O.C	Number	Nearest_neighbor
HMWL 0h	0	1.592361	0.688883	585	Levulinic acid
HMWL 0h	0	1.588208	0.326024	718	Cyclopentanone
HMWL 0h	0	0.786368	0.287091	1187	Furfural
HMWL 0h	0	1.010343	0.340461	2278	Benzenediol
HMWL 0h	0	1.267679	0.531225	1535	5-HMF
HMWL 0h	0	1.235375	0.290024	1548	Coniferyl alcohol
HMWL 0.5h	0.5	1.310744	0.311826	1532	Sinapyl alcohol
HMWL 0.5h	0.5	1.158936	0.505987	1295	Humin
HMWL 0.5h	0.5	0.803046	0.297576	1363	Furfural
HMWL 0.5h	0.5	1.518778	0.611708	691	Levulinic acid
HMWL 0.5h	0.5	1.688423	0.272787	560	Cyclopentanone
HMWL 0.5h	0.5	1.0402	0.289795	2157	Benzenediol
HMWL 1h	1	1.018999	0.343378	2446	Benzenediol
HMWL 1h	1	0.779715	0.288511	1278	Furfural
HMWL 1h	1	1.305129	0.542288	1548	Fulvic acid
HMWL 1h	1	1.259607	0.293457	1641	Sinapyl alcohol
HMWL 1h	1	1.64967	0.289965	731	Cyclopentanone
HMWL 1h	1	1.661485	0.735957	545	Levoglucosan

HMWL 2h	2	1.501341	0.656533	671	Levulinic acid
HMWL 2h	2	1.0573	0.29025	2047	Benzenediol
HMWL 2h	2	1.302629	0.35483	1516	Sinapyl alcohol
HMWL 2h	2	1.136191	0.520943	1184	Humic acid
HMWL 2h	2	1.62801	0.32955	478	Cyclopentanone
HMWL 2h	2	0.826011	0.314389	1272	Furfural
HMWL 4h	4	1.049432	0.287181	1841	Benzenediol
HMWL 4h	4	0.820258	0.299609	1229	Furfural
HMWL 4h	4	1.489625	0.619547	736	Levulinic acid
HMWL 4h	4	1.142092	0.506562	1182	Humic acid
HMWL 4h	4	1.30553	0.324724	1390	Sinapyl alcohol
HMWL 4h	4	1.662569	0.291558	525	Cyclopentanone
LMWL 0h	0	1.492104	0.55223	867	Butanedial
LMWL 0h	0	1.355071	0.26073	1744	Dimethoxyphenol
LMWL 0h	0	1.695925	0.227523	901	Cyclopentanone
LMWL 0h	0	1.087831	0.236504	1879	Coumaryl alcohol
LMWL 0h	0	1.157175	0.462261	1349	Humin
LMWL 0h	0	0.844466	0.285536	1609	Furfural
LMWL 0.5h	0.5	1.090428	0.235724	2131	Coumaryl alcohol
LMWL 0.5h	0.5	1.692058	0.231135	1018	Cyclopentanone
LMWL 0.5h	0.5	1.177686	0.451024	1475	Humin
LMWL 0.5h	0.5	1.363914	0.248617	1850	Dehydroabietic acid
LMWL 0.5h	0.5	0.845856	0.274146	1697	Furfural
LMWL 0.5h	0.5	1.504965	0.550889	844	Butanedial
LMWL 1 h	1	1.534443	0.665454	570	Levulinic acid
LMWL 1 h	1	1.229079	0.470836	1300	Humin
LMWL 1 h	1	1.383631	0.256376	1477	Dehydroabietic acid
LMWL 1 h	1	0.846677	0.250659	1320	Furfural
LMWL 1 h	1	1.093843	0.262753	2236	Coumaryl alcohol
LMWL 1 h	1	0.812322	0.631681	520	Maeylacetic acid
LMWL 1 h	1	1.737857	0.248763	869	Cyclopentanone
LMWL 2h	2	1.115124	0.485793	1127	Humic acid
LMWL 2h	2	1.348669	0.275006	1769	Dimethoxyphenol
LMWL 2h	2	0.828494	0.270368	1260	Furfural
LMWL 2h	2	1.083894	0.237485	1766	Coumaryl alcohol
LMWL 2h	2	1.486544	0.582445	748	Butanedial
LMWL 2h	2	1.698354	0.235349	860	Cyclopentanone
LMWL 4h	4	1.187055	0.466368	1379	Humin
LMWL 4h	4	1.348942	0.263837	1736	Dimethoxyphenol
LMWL 4h	4	1.691602	0.231913	834	Cyclopentanone
LMWL 4h	4	0.825141	0.266131	1305	Furfural
LMWL 4h	4	1.52513	0.586321	766	Levulinic acid
LMWL 4h	4	1.077299	0.253189	2227	Coumaryl alcohol
LMWL 6h	6	0.83322	0.271863	1652	Furfural
LMWL 6h	6	1.707166	0.230809	1007	Cyclopentanone
LMWL 6h	6	1.361759	0.245393	1924	Dehydroabietic acid

LMWL 6h	6	1.185509	0.455547	1505	Humin
LMWL 6h	6	1.082862	0.239344	2344	Coumaryl alcohol
LMWL 6h	6	1.517564	0.550492	878	Butanedial
LMWL 11h	11	1.482635	0.539777	859	Butanedial
LMWL 11h	11	1.100887	0.231701	1837	Coumaryl alcohol
LMWL 11h	11	1.161421	0.452325	1350	Humin
LMWL 11h	11	1.684795	0.231526	894	Cyclopentanone
LMWL 11h	11	1.358081	0.256568	1723	Dehydroabietic acid
LMWL 11h	11	0.86371	0.277507	1600	Furfural
LMWL 17h	17	1.344185	0.248282	1648	Dehydroabietic acid
LMWL 17h	17	0.861416	0.280848	1517	Furfural
LMWL 17h	17	1.087744	0.24115	1941	Coumaryl alcohol
LMWL 17h	17	1.483797	0.529074	807	Butanedial
LMWL 17h	17	1.166187	0.447062	1362	Humin
LMWL 17h	17	1.683961	0.231368	863	Cyclopentanone
LMWL 24h	24	0.841886	0.282151	1695	Furfural
LMWL 24h	24	1.091194	0.23181	2043	Coumaryl alcohol
LMWL 24h	24	1.157874	0.463149	1455	Humin
LMWL 24h	24	1.360717	0.262774	1905	Dehydroabietic acid
LMWL 24h	24	1.692746	0.232249	930	Cyclopentanone
LMWL 24h	24	1.50111	0.571499	858	Butanedial
LMWL 41h	41	1.689556	0.237421	830	Cyclopentanone
LMWL 41h	41	1.345373	0.255426	1797	Sinapyl alcohol
LMWL 41h	41	0.836106	0.284	1567	Furfural
LMWL 41h	41	1.154682	0.448418	1458	Humin
LMWL 41h	41	1.076451	0.240828	2087	Coumaryl alcohol
LMWL 41h	41	1.474579	0.544287	843	Butanedial
WS 0h	0	1.634614	0.319507	641	Cyclopentanone
WS 0h	0	1.086167	0.525511	1060	Humic acid
WS 0h	0	1.199993	0.310778	1558	Coniferyl alcohol
WS 0h	0	1.643256	0.73617	797	Levoglucosan
WS 0h	0	1.380061	0.570322	1437	Butanedial
WS 0h	0	0.894423	0.302147	1529	Furfural
WS 0.5h	0.5	1.633402	0.73713	913	Levoglucosan
WS 0.5h	0.5	1.378874	0.573608	1526	Butanedial
WS 0.5h	0.5	1.203872	0.328962	1467	Coniferyl alcohol
WS 0.5h	0.5	0.910863	0.313914	1429	Furfural
WS 0.5h	0.5	1.65214	0.33262	568	Cyclopentanone
WS 0.5h	0.5	1.098784	0.524834	1085	Humic acid
WS 1h	1	1.655418	0.325705	584	Cyclopentanone
WS 1h	1	1.204213	0.322367	1456	Coniferyl alcohol
WS 1h	1	1.655476	0.73749	818	Levoglucosan
WS 1h	1	0.907969	0.31626	1477	Furfural
WS 1h	1	1.10466	0.527336	1102	Humic acid
WS 1h	1	1.388126	0.574578	1530	Butanedial
WS 2h	2	1.642439	0.741077	882	Levoglucosan

WS 2h	2	1.102754	0.520276	998	Humic acid
WS 2h	2	1.37541	0.60089	1333	Fulvic acid
WS 2h	2	1.583193	0.383378	518	Levulinic acid
WS 2h	2	1.213742	0.346779	1346	Coniferyl alcohol
WS 2h	2	0.925405	0.316685	1393	Furfural
WS 4h	4	1.594174	0.386876	550	Levulinic acid
WS 4h	4	1.08239	0.518966	926	Humic acid
WS 4h	4	1.207829	0.334088	1262	Coniferyl alcohol
WS 4h	4	1.367486	0.601696	1182	Fulvic acid
WS 4h	4	1.651092	0.744493	781	Levoglucosan
WS 4h	4	0.902657	0.315662	1204	Furfural
WS 6h	6	1.381883	0.583952	1423	Butanedial
WS 6h	6	1.653667	0.743498	814	Levoglucosan
WS 6h	6	1.193298	0.337722	1432	Coniferyl alcohol
WS 6h	6	1.08588	0.533554	989	Humic acid
WS 6h	6	0.896272	0.313113	1335	Furfural
WS 6h	6	1.618879	0.342098	520	Cyclopentanone
WS 11h	11	1.618784	0.726947	917	Levoglucosan
WS 11h	11	1.294551	0.624814	1093	Fulvic acid
WS 11h	11	1.416822	0.361861	992	Coniferyl alcohol
WS 11h	11	1.753542	0.283145	726	Oleic acid
WS 11h	11	1.116648	0.382823	1662	Humic acid
WS 11h	11	0.765022	0.639542	470	Maleylacetic acid
WS 11h	11	0.882367	0.294931	912	Furfural
WS 24h	24	1.573349	0.374058	535	Levulinic acid
WS 24h	24	0.901071	0.325962	1402	Furfural
WS 24h	24	1.645117	0.730729	891	Levoglucosan
WS 24h	24	1.188624	0.345602	1544	Coniferyl alcohol
WS 24h	24	1.086937	0.527503	1117	Humic acid
WS 24h	24	1.36547	0.593291	1542	Fulvic acid
WS 34h	34	1.63621	0.727398	896	Levoglucosan
WS 34h	34	1.627744	0.345246	532	Cyclopentanone
WS 34h	34	1.183347	0.327386	1518	Coniferyl alcohol
WS 34h	34	0.878855	0.317177	1390	Furfural
WS 34h	34	1.086294	0.522103	1127	Humic acid
WS 34h	34	1.370241	0.577644	1530	Fulvic acid
WS 50h	50	1.18102	0.335898	1455	Coniferyl alcohol
WS 50h	50	1.086573	0.523852	1121	Humic acid
WS 50h	50	1.363902	0.581559	1552	Fulvic acid
WS 50h	50	1.633024	0.733246	892	Levoglucosan
WS 50h	50	1.599683	0.352276	503	Cyclopentanone
WS 50h	50	0.882655	0.32685	1243	Furfural

id_general	id	hours	H.C	O.C	Number	Nearest_neighbor

HMWL	HMWL 0h	0	1.592361	0.688883	585	Levulinic acid
HMWL	HMWL 0h	0	1.588208	0.326024	718	Cyclopentanone
HMWL	HMWL 0h	0	0.786368	0.287091	1187	Furfural
HMWL	HMWL 0h	0	1.010343	0.340461	2278	Benzenediol
HMWL	HMWL 0h	0	1.267679	0.531225	1535	5-HMF
HMWL	HMWL 0h	0	1.235375	0.290024	1548	Coniferyl alcohol
HMWL	HMWL 0.5h	0.5	1.310744	0.311826	1532	Sinapyl alcohol
HMWL	HMWL 0.5h	0.5	1.158936	0.505987	1295	Humin
HMWL	HMWL 0.5h	0.5	0.803046	0.297576	1363	Furfural
HMWL	HMWL 0.5h	0.5	1.518778	0.611708	691	Levulinic acid
HMWL	HMWL 0.5h	0.5	1.688423	0.272787	560	Cyclopentanone
HMWL	HMWL 0.5h	0.5	1.0402	0.289795	2157	Benzenediol
HMWL	HMWL 1h	1	1.018999	0.343378	2446	Benzenediol
HMWL	HMWL 1h	1	0.779715	0.288511	1278	Furfural
HMWL	HMWL 1h	1	1.305129	0.542288	1548	Fulvic acid
HMWL	HMWL 1h	1	1.259607	0.293457	1641	Sinapyl alcohol
HMWL	HMWL 1h	1	1.64967	0.289965	731	Cyclopentanone
HMWL	HMWL 1h	1	1.661485	0.735957	545	Levoglucosan
HMWL	HMWL 2h	2	1.501341	0.656533	671	Levulinic acid
HMWL	HMWL 2h	2	1.0573	0.29025	2047	Benzenediol
HMWL	HMWL 2h	2	1.302629	0.35483	1516	Sinapyl alcohol
HMWL	HMWL 2h	2	1.136191	0.520943	1184	Humic acid
HMWL	HMWL 2h	2	1.62801	0.32955	478	Cyclopentanone
HMWL	HMWL 2h	2	0.826011	0.314389	1272	Furfural
HMWL	HMWL 4h	4	1.049432	0.287181	1841	Benzenediol
HMWL	HMWL 4h	4	0.820258	0.299609	1229	Furfural
HMWL	HMWL 4h	4	1.489625	0.619547	736	Levulinic acid

HMWL	HMWL 4h	4	1.142092	0.506562	1182	Humic acid
HMWL	HMWL 4h	4	1.30553	0.324724	1390	Sinapyl alcohol
HMWL	HMWL 4h	4	1.662569	0.291558	525	Cyclopentanone
LMWL	LMWL 0h	0	1.492104	0.55223	867	Butanedial
LMWL	LMWL 0h	0	1.355071	0.26073	1744	Dimethoxyphenol
LMWL	LMWL 0h	0	1.695925	0.227523	901	Cyclopentanone
LMWL	LMWL 0h	0	1.087831	0.236504	1879	Coumaryl alcohol
LMWL	LMWL 0h	0	1.157175	0.462261	1349	Humin
LMWL	LMWL 0h	0	0.844466	0.285536	1609	Furfural
LMWL	LMWL 0.5h	0.5	1.090428	0.235724	2131	Coumaryl alcohol
LMWL	LMWL 0.5h	0.5	1.692058	0.231135	1018	Cyclopentanone
LMWL	LMWL 0.5h	0.5	1.177686	0.451024	1475	Humin
LMWL	LMWL 0.5h	0.5	1.363914	0.248617	1850	Dehydroabietic acid
LMWL	LMWL 0.5h	0.5	0.845856	0.274146	1697	Furfural
LMWL	LMWL 0.5h	0.5	1.504965	0.550889	844	Butanedial
LMWL	LMWL 1 h	1	1.534443	0.665454	570	Levulinic acid
LMWL	LMWL 1 h	1	1.229079	0.470836	1300	Humin
LMWL	LMWL 1 h	1	1.383631	0.256376	1477	Dehydroabietic acid
LMWL	LMWL 1 h	1	0.846677	0.250659	1320	Furfural
LMWL	LMWL 1 h	1	1.093843	0.262753	2236	Coumaryl alcohol
LMWL	LMWL 1 h	1	0.812322	0.631681	520	Maeylacetic acid
LMWL	LMWL 1 h	1	1.737857	0.248763	869	Cyclopentanone
LMWL	LMWL 2h	2	1.115124	0.485793	1127	Humic acid
LMWL	LMWL 2h	2	1.348669	0.275006	1769	Dimethoxyphenol
LMWL	LMWL 2h	2	0.828494	0.270368	1260	Furfural
LMWL	LMWL 2h	2	1.083894	0.237485	1766	Coumaryl alcohol
LMWL	LMWL 2h	2	1.486544	0.582445	748	Butanedial

LMWL	LMWL 2h	2	1.698354	0.235349	860	Cyclopentanone
LMWL	LMWL 4h	4	1.187055	0.466368	1379	Humin
LMWL	LMWL 4h	4	1.348942	0.263837	1736	Dimethoxyphenol
LMWL	LMWL 4h	4	1.691602	0.231913	834	Cyclopentanone
LMWL	LMWL 4h	4	0.825141	0.266131	1305	Furfural
LMWL	LMWL 4h	4	1.52513	0.586321	766	Levulinic acid
LMWL	LMWL 4h	4	1.077299	0.253189	2227	Coumaryl alcohol
LMWL	LMWL 6h	6	0.83322	0.271863	1652	Furfural
LMWL	LMWL 6h	6	1.707166	0.230809	1007	Cyclopentanone
LMWL	LMWL 6h	6	1.361759	0.245393	1924	Dehydroabietic acid
LMWL	LMWL 6h	6	1.185509	0.455547	1505	Humin
LMWL	LMWL 6h	6	1.082862	0.239344	2344	Coumaryl alcohol
LMWL	LMWL 6h	6	1.517564	0.550492	878	Butanedial
LMWL	LMWL 11h	11	1.482635	0.539777	859	Butanedial
LMWL	LMWL 11h	11	1.100887	0.231701	1837	Coumaryl alcohol
LMWL	LMWL 11h	11	1.161421	0.452325	1350	Humin
LMWL	LMWL 11h	11	1.684795	0.231526	894	Cyclopentanone
LMWL	LMWL 11h	11	1.358081	0.256568	1723	Dehydroabietic acid
LMWL	LMWL 11h	11	0.86371	0.277507	1600	Furfural
LMWL	LMWL 17h	17	1.344185	0.248282	1648	Dehydroabietic acid
LMWL	LMWL 17h	17	0.861416	0.280848	1517	Furfural
LMWL	LMWL 17h	17	1.087744	0.24115	1941	Coumaryl alcohol
LMWL	LMWL 17h	17	1.483797	0.529074	807	Butanedial
LMWL	LMWL 17h	17	1.166187	0.447062	1362	Humin
LMWL	LMWL 17h	17	1.683961	0.231368	863	Cyclopentanone
LMWL	LMWL 24h	24	0.841886	0.282151	1695	Furfural
LMWL	LMWL 24h	24	1.091194	0.23181	2043	Coumaryl alcohol

LMWL	LMWL 24h	24	1.157874	0.463149	1455	Humin
LMWL	LMWL 24h	24	1.360717	0.262774	1905	Dehydroabietic acid
LMWL	LMWL 24h	24	1.692746	0.232249	930	Cyclopentanone
LMWL	LMWL 24h	24	1.50111	0.571499	858	Butanedial
LMWL	LMWL 41h	41	1.689556	0.237421	830	Cyclopentanone
LMWL	LMWL 41h	41	1.345373	0.255426	1797	Sinapyl alcohol
LMWL	LMWL 41h	41	0.836106	0.284	1567	Furfural
LMWL	LMWL 41h	41	1.154682	0.448418	1458	Humin
LMWL	LMWL 41h	41	1.076451	0.240828	2087	Coumaryl alcohol
LMWL	LMWL 41h	41	1.474579	0.544287	843	Butanedial
H2O	H2O 0h	0	1.634614	0.319507	641	Cyclopentanone
H2O	H2O 0h	0	1.086167	0.525511	1060	Humic acid
H2O	H2O 0h	0	1.199993	0.310778	1558	Coniferyl alcohol
H2O	H2O 0h	0	1.643256	0.73617	797	Levoglucosan
H2O	H2O 0h	0	1.380061	0.570322	1437	Butanedial
H2O	H2O 0h	0	0.894423	0.302147	1529	Furfural
H2O	H2O 0.5h	0.5	1.633402	0.73713	913	Levoglucosan
H2O	H2O 0.5h	0.5	1.378874	0.573608	1526	Butanedial
H2O	H2O 0.5h	0.5	1.203872	0.328962	1467	Coniferyl alcohol
H2O	H2O 0.5h	0.5	0.910863	0.313914	1429	Furfural
H2O	H2O 0.5h	0.5	1.65214	0.33262	568	Cyclopentanone
H2O	H2O 0.5h	0.5	1.098784	0.524834	1085	Humic acid
H2O	H2O 1h	1	1.655418	0.325705	584	Cyclopentanone
H2O	H2O 1h	1	1.204213	0.322367	1456	Coniferyl alcohol
H2O	H2O 1h	1	1.655476	0.73749	818	Levoglucosan
H2O	H2O 1h	1	0.907969	0.31626	1477	Furfural
H2O	H2O 1h	1	1.10466	0.527336	1102	Humic acid
H2O	H2O 1h	1	1.388126	0.574578	1530	Butanedial
H2O	H2O 2h	2	1.642439	0.741077	882	Levoglucosan
H2O	H2O 2h	2	1.102754	0.520276	998	Humic acid
H2O	H2O 2h	2	1.37541	0.60089	1333	Fulvic acid
H2O	H2O 2h	2	1.583193	0.383378	518	Levulinic acid
H2O	H2O 2h	2	1.213742	0.346779	1346	Coniferyl alcohol
H2O	H2O 2h	2	0.925405	0.316685	1393	Furfural

H2O	H2O 4h	4	1.594174	0.386876	550	Levulinic acid
H2O	H2O 4h	4	1.08239	0.518966	926	Humic acid
H2O	H2O 4h	4	1.207829	0.334088	1262	Coniferyl alcohol
H2O	H2O 4h	4	1.367486	0.601696	1182	Fulvic acid
H2O	H2O 4h	4	1.651092	0.744493	781	Levoglucosan
H2O	H2O 4h	4	0.902657	0.315662	1204	Furfural
H2O	H2O 6h	6	1.381883	0.583952	1423	Butanedial
H2O	H2O 6h	6	1.653667	0.743498	814	Levoglucosan
H2O	H2O 6h	6	1.193298	0.337722	1432	Coniferyl alcohol
H2O	H2O 6h	6	1.08588	0.533554	989	Humic acid
H2O	H2O 6h	6	0.896272	0.313113	1335	Furfural
H2O	H2O 6h	6	1.618879	0.342098	520	Cyclopentanone
H2O	H2O 11h	11	1.618784	0.726947	917	Levoglucosan
H2O	H2O 11h	11	1.294551	0.624814	1093	Fulvic acid
H2O	H2O 11h	11	1.416822	0.361861	992	Coniferyl alcohol
H2O	H2O 11h	11	1.753542	0.283145	726	Oleic acid
H2O	H2O 11h	11	1.116648	0.382823	1662	Humic acid
H2O	H2O 11h	11	0.765022	0.639542	470	Maleylacetic acid
H2O	H2O 11h	11	0.882367	0.294931	912	Furfural
H2O	H2O 24h	24	1.573349	0.374058	535	Levulinic acid
H2O	H2O 24h	24	0.901071	0.325962	1402	Furfural
H2O	H2O 24h	24	1.645117	0.730729	891	Levoglucosan
H2O	H2O 24h	24	1.188624	0.345602	1544	Coniferyl alcohol
H2O	H2O 24h	24	1.086937	0.527503	1117	Humic acid
H2O	H2O 24h	24	1.36547	0.593291	1542	Fulvic acid
H2O	H2O 34h	34	1.63621	0.727398	896	Levoglucosan
H2O	H2O 34h	34	1.627744	0.345246	532	Cyclopentanone
H2O	H2O 34h	34	1.183347	0.327386	1518	Coniferyl alcohol
H2O	H2O 34h	34	0.878855	0.317177	1390	Furfural
H2O	H2O 34h	34	1.086294	0.522103	1127	Humic acid
H2O	H2O 34h	34	1.370241	0.577644	1530	Fulvic acid
H2O	H2O 50h	50	1.18102	0.335898	1455	Coniferyl alcohol

H2O	H2O 50h	50	1.086573	0.523852	1121	Humic acid
H2O	H2O 50h	50	1.363902	0.581559	1552	Fulvic acid
H2O	H2O 50h	50	1.633024	0.733246	892	Levoglucosan
H2O	H2O 50h	50	1.599683	0.352276	503	Cyclopentanone
H2O	H2O 50h	50	0.882655	0.32685	1243	Furfural

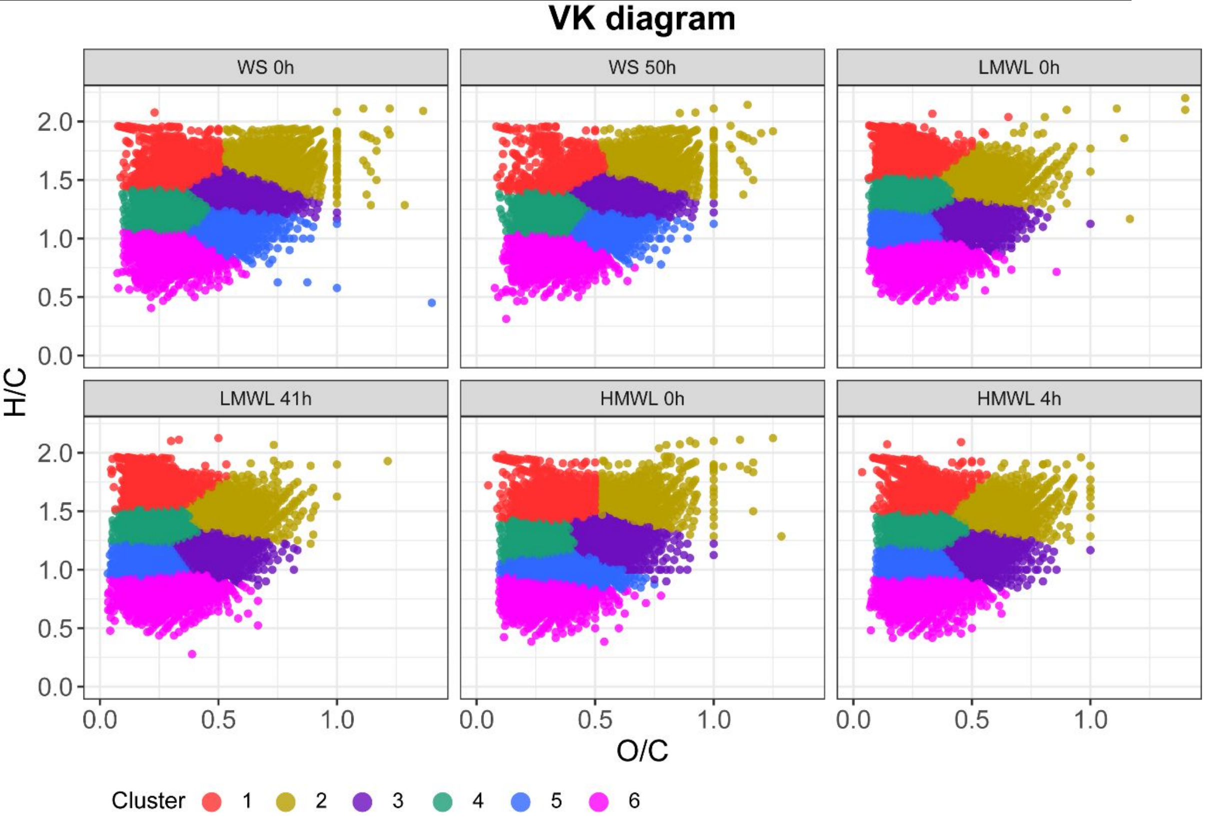

Figure S 8. Van Krevelen diagrams illustrating the k-means clustering of the raw samples and their last oxidation point.

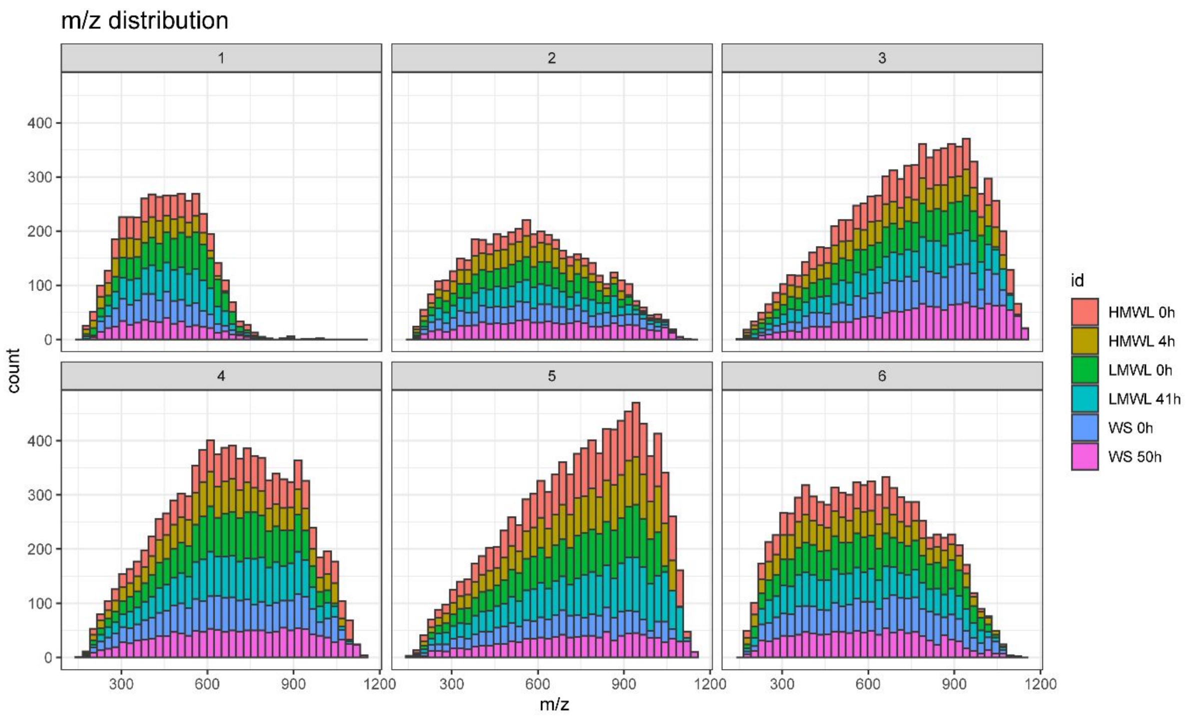

Figure S 9. m/z distribution per cluster of the fractions WS, LWML and HMWL at two different oxidation points.

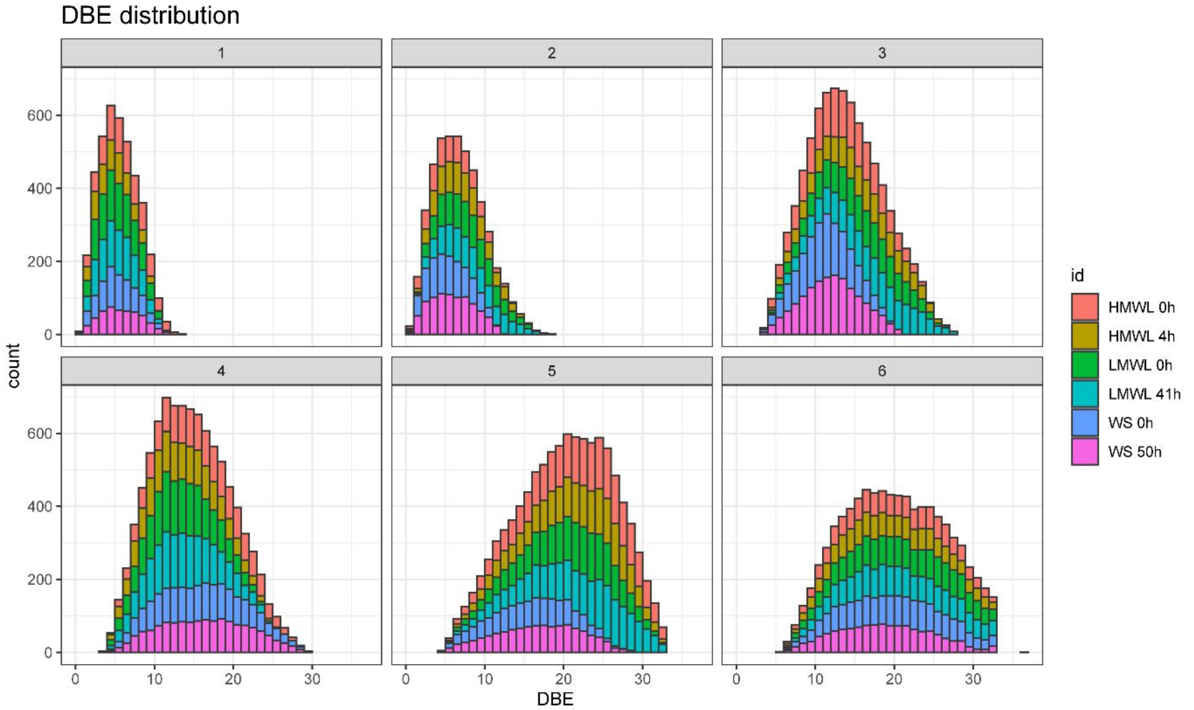

Figure S 10. Distribution of the double bond equivalent per cluster of the fractions WS, LWML and HMWL before accelerated aging and after the highest acceleration aging point.

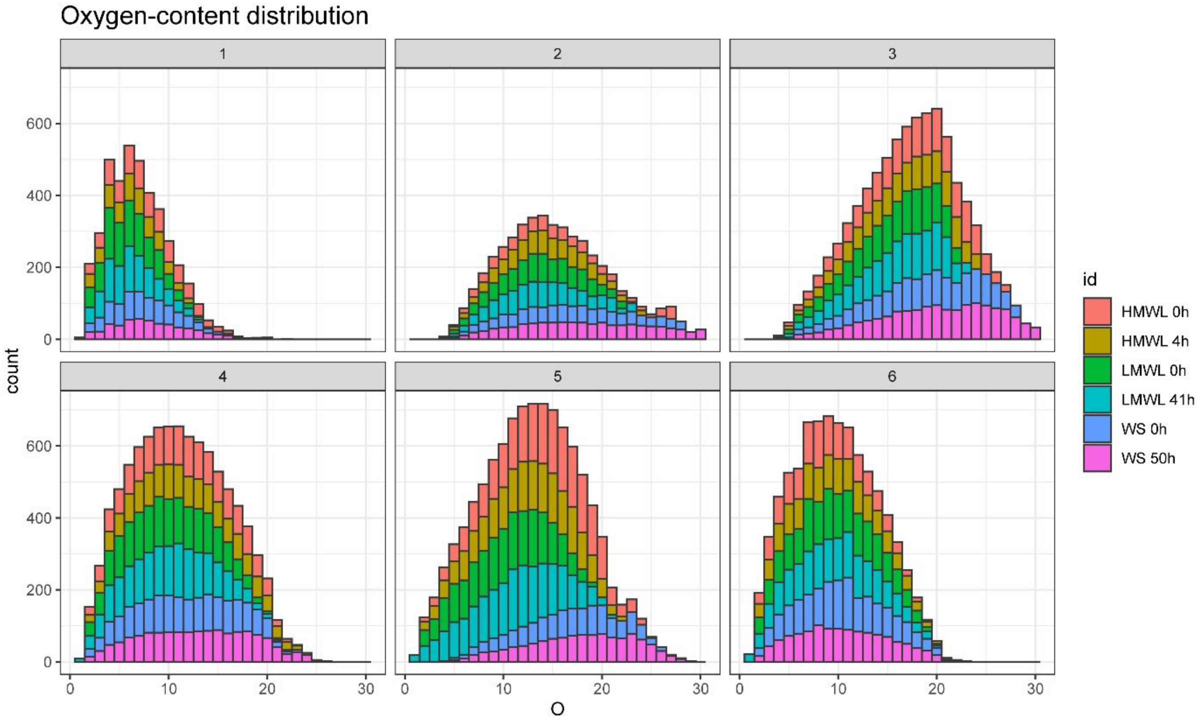

Figure S 11 Distribution of the oxygen content per cluster of the fractions WS, LWML and HMWL before accelerated aging and after the highest acceleration aging point.

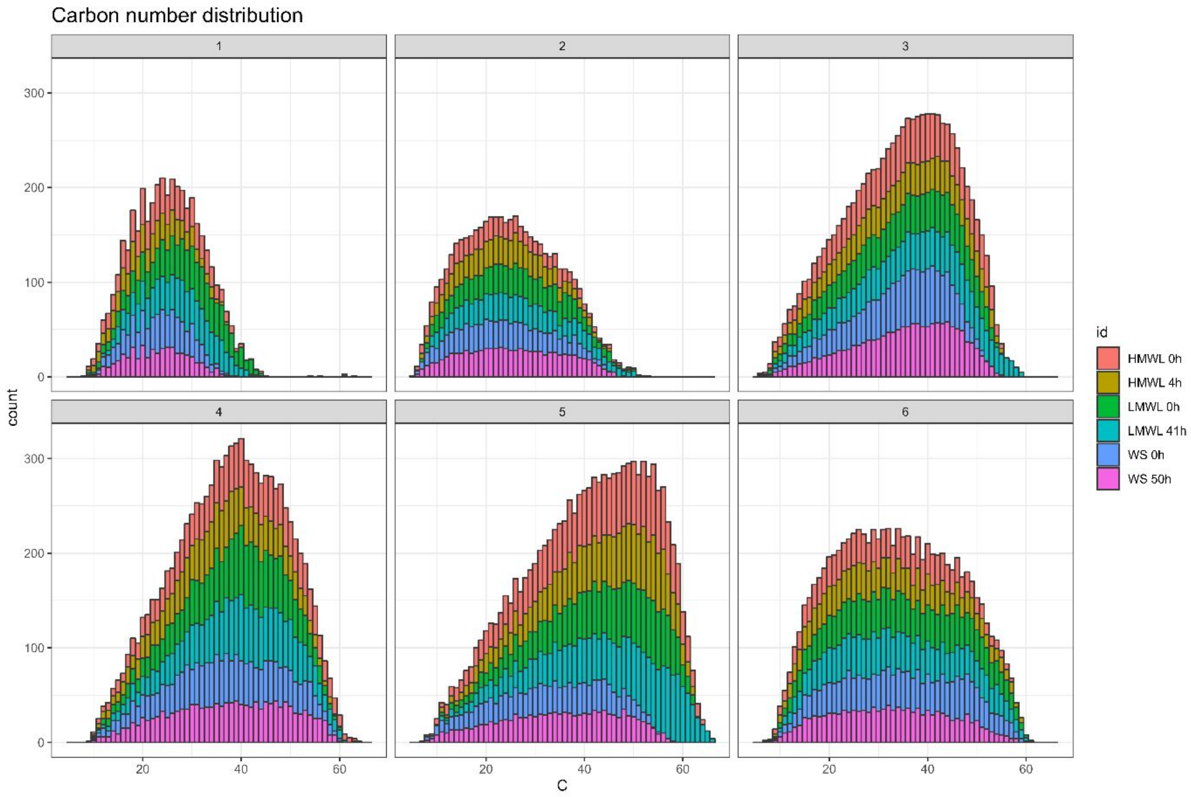

Figure S 12. C Distribution of the carbon number per cluster of the fractions WS, LWML and HMWL before accelerated aging and after the highest acceleration aging point.

Reference

(1) Rice, J. A.; MacCarthy, P. Statistical Evaluation of the Elemental Composition of Humic Substances. *Org Geochem* **1991**, *17* (5), 635–648. https://doi.org/10.1016/0146-6380(91)90006-6.
